# The prognostic value of systemic immune-inflammation index in patients with unresectable hepatocellular carcinoma treated with immune-based therapy

**DOI:** 10.1186/s40364-024-00722-6

**Published:** 2025-01-14

**Authors:** Tian He, Bin Xu, Lu-Na Wang, Zi-Yi Wang, Huan-Chen Shi, Cheng-Jie Zhong, Xiao-Dong Zhu, Ying-Hao Shen, Jian Zhou, Jia Fan, Hui-Chuan Sun, Bo Hu, Cheng Huang

**Affiliations:** Department of Liver Surgery and Transplantation, Liver Cancer Institute and Zhongshan Hospital, Fudan University180 Fenglin Road, Shanghai, 200032 China

**Keywords:** Hepatocellular carcinoma, Immune-based therapy, Atezolizumab-bevacizumab, Systemic immune-inflammation index (SII), Prognosis

## Abstract

**Background:**

Predicting the efficacy of immune-based therapy in patients with unresectable hepatocellular carcinoma (HCC) remains a clinical challenge. This study aims to evaluate the prognostic value of the systemic immune-inflammation index (SII) in forecasting treatment response and survival outcomes for HCC patients undergoing immune-based therapy.

**Methods:**

We analyzed a cohort of 268 HCC patients treated with immune-based therapy from January 2019 to March 2023. A training cohort of 93 patients received atezolizumab plus bevacizumab (T + A), while a validation cohort of 175 patients underwent treatment with tyrosine kinase inhibitors (TKIs) combined with anti-PD-(L)1 therapy. The SII cutoff value, determined using X-tile analysis based on overall survival (OS) in the training cohort, divided patients into high (> 752*10^9^) and low (≤ 752*10^9^) SII groups. Prognostic factors were identified through univariate and multivariate logistic and Cox regression analyses, and survival outcomes were assessed using Kaplan–Meier methods. The predictive accuracy of SII was evaluated using receiver operating characteristic (ROC) curves.

**Results:**

An optimal SII cutoff of 752*10^9^ stratified patients into high and low SII groups. Univariate and multivariate logistic regression indicated that SII was a significant predictor of the objective response rate (ORR), which was markedly different between the low and high SII subgroups (34.72% vs. 9.52%, *P* = 0.019). This finding was consistent in the validation cohort (34.09% vs. 16.28%, *P* = 0.026). SII also demonstrated prognostic value in Cox regression and Kaplan–Meier analyses. ROC curves confirmed that SII had superior predictive accuracy compared to common clinical indicators, with predictive relevance even in AFP-negative patients. Furthermore, a lower SII was associated with a higher T cell ratio and an increased number of CD8^+^ T cells and Granzyme B^+^ CD8^+^ T cells in peripheral blood.

**Conclusion:**

SII is a promising predictor of both therapeutic efficacy and prognosis in HCC patients undergoing immune-based treatments. Its application may enhance clinical decision-making, thereby improving patient outcomes from immune-based therapy.

**Supplementary Information:**

The online version contains supplementary material available at 10.1186/s40364-024-00722-6.

## Introduction

Primary liver cancer is the sixth most common cancer worldwide, and its incidence is increasing [1]. HCC, the primary type of liver cancer, typically develops in the context of chronic liver disease. Common causes include hepatitis B virus (HBV), hepatitis C virus (HCV) infection, alcohol abuse, or factors related to metabolic syndrome [2]. For early-stage HCC patients, curative treatments include surgical resection, transarterial chemoembolization (TACE), radio-frequency ablation, and liver transplantation [3]. In more advanced stages, immune checkpoint inhibitors (ICIs) targeting the programmed cell death protein 1 (PD-1) and its ligand PD-L1 have become promising treatments [4]. Atezolizumab and bevacizumab combination therapy, in particular, has shown significant effectiveness and safety for advanced-stage HCC, and is now recommended as a first-line treatment [5].

However, the absence of reliable predictive biomarkers for immune-based therapy remains a major challenge. Although tumor tissue biomarkers like tumor PD-L1 expression, tumor mutational burden (TMB), mismatch repair protein (MMR), and microsatellite instability (MSI) are promising, they are limited by the tumor microenvironment’s complexity and their lack of specificity and accuracy [6, 7]. These biomarkers are also expensive and not routinely available. As a result, regular hematological markers associated with inflammation are gaining attention [8–10]. The systemic immune-inflammatory response is crucial in HCC development and progression [11]. This is highlighted by the significance of systemic inflammation markers such as the neutrophil-to-lymphocyte ratio (NLR) and the platelet-to-lymphocyte ratio (PLR). High levels of NLR or PLR in HCC indicate a pro-inflammatory tumor environment [12] and are linked to poorer survival outcomes in patients receiving T + A treatment [13, 14].

Our research group previously introduced the SII score as an innovative biomarker for assessing recurrence risk in HCC post-resection [15]. Initially proposed in our earlier studies, SII has proven its prognostic value in various cancers, including small cell lung cancer, gastroesophageal adenocarcinoma, and esophageal squamous cell carcinoma [15–17]. SII’s non-invasive nature and accessibility through routine blood tests make it a promising tool for predicting immune-based therapy effectiveness in HCC. This study aims to further explore SII’s role in guiding therapeutic decisions for HCC patients, particularly in immune-based treatments, offering a significant contribution to precision oncology.

## Patients and methods

### Patients and specimens

This study included 268 HCC patients from Zhongshan Hospital, Fudan University, China, who received immune-based therapy from January 2019 to March 2023. The detailed dosages for the different immune-based strategies were as follows: bevacizumab or its biosimilar IBI305 at a dose of 15 mg/kg every 3 weeks, rivoceranib 250 mg once daily, or lenvatinib 8 mg/day. The anti-PD(L)-1 antibodies included atezolizumab 1200 mg every 3 weeks, nivolumab 3 mg/kg every 2 weeks, camrelizumab 200 mg every 2 weeks, pembrolizumab 200 mg every 3 weeks, sintilimab 200 mg every 3 weeks, or toripalimab 240 mg every 3 weeks. Most of the patients were over 50 years old (68.28%). The cohort predominantly comprised males (66.42%). Inclusion criteria were: 1) clinical symptoms, imaging features, and serological molecular markers aligning with HCC diagnostic criteria [18]; 2) receipt of at least two courses of anti-PD-(L)1 treatment. Exclusion criteria included: 1) incomplete hematological data; 2) lack of follow-up; 3) receipt of locoregional therapy (e.g., TACE, radiofrequency ablation) at baseline or surgery post immune-based therapy. Considering the prominence of T + A treatment as first-line therapy for unresectable HCC, we assigned 93 patients (from October 2020 to March 2023) receiving this treatment as the training cohort. To mirror real-world immune-based therapy, 175 patients (from January 2019 to December 2021) who received TKIs plus anti-PD-(L)1 therapy were included as the validation cohort. Baseline blood samples for research were collected prior to immune-based therapy initiation and analyzed in the laboratory department.

Tumor stages were assessed using the Barcelona Clinic Liver Cancer (BCLC) staging system [19]. Liver function was evaluated by the Child–Pugh scoring system. Treatment response was measured using the Response Evaluation Criteria in Solid Tumors V.1.1 (RECIST V.1.1). Objective Response Rate (ORR) was defined as the proportion of patients achieving complete response (CR) or partial response (PR). Patients with CR or PR were considered responders, while those with stable disease (SD) or progressive disease (PD) were non-responders. Overall survival (OS) was calculated from the first cycle of immune-based therapy to death due to any cause, or censored on the last follow-up, and progression-free survival (PFS) was measured from the first cycle to the occurrence of disease progression, death from any cause; or censored on the last follow-up.

The study was conducted in accordance with the Declaration of Helsinki and written informed consent was obtained from all patients before inclusion in the study. The study protocol was approved by the Ethics Committee of Zhongshan Hospital (Approval Number B2020-177R) and followed the Declaration of Helsinki.

### Data collection and follow-up

Data collected via an electronic medical record system encompassed patient baseline information (age, gender, etc.), laboratory test results (complete blood count, AFP, ALT, etc.), immune-based therapy related data (baseline time, treatment strategy, response state, etc.), and tumor-related information (CNLC, extrahepatic metastasis, etc.). Follow-ups were conducted every 60 days (± 7 days) after the initiation of combination therapy until October 2023 for both cohorts.

### Immune-based prognostic scores

The Systemic Immune-Inflammation Index (SII) was calculated using the formula: SII = Platelet count * Neutrophil count / Lymphocyte count, as previously established [15]. The training cohort data was analyzed using X-tile 3.6.1 software (Yale University, New Haven, CT) to determine the optimal SII cutoff for overall survival (Supplementary Figure. S1) [20]. NLR and PLR were calculated as Neutrophil count / Lymphocyte count and Platelet count / Lymphocyte count, respectively, with previously defined cutoff values [15, 21].

### Statistical analysis

Statistical analyses were conducted using IBM SPSS software (version 29.0; IBM Corp., Armonk, NY). Continuous variables were tested for normality with the Shapiro–Wilk test and compared using the t-test or Mann–Whitney U test. They were summarized as median and range or mean ± SEM. Categorical variables, presented as counts and percentages, were compared using Fisher’s exact test or Pearson’s χ2 test. OS and PFS were calculated using the Kaplan–Meier method, with differences between groups assessed by the log-rank test. Univariate and multivariate logistic regression identified independent predictors. Univariate and multivariate Cox regression analyses determined risk factors for OS and PFS. Variables with a P value < 0.1 in univariate analyses were included in multivariate analyses [22]. Receiver Operating Characteristic (ROC) curves were used to evaluate sensitivity and specificity, and differences in the area under the curves (AUC) were analyzed by MedCalc version 13.0 (MedCalc Software) using Delong test. A P value < 0.05 was considered statistically significant.

## Results

### Patient characteristics

**Fig. 1:**
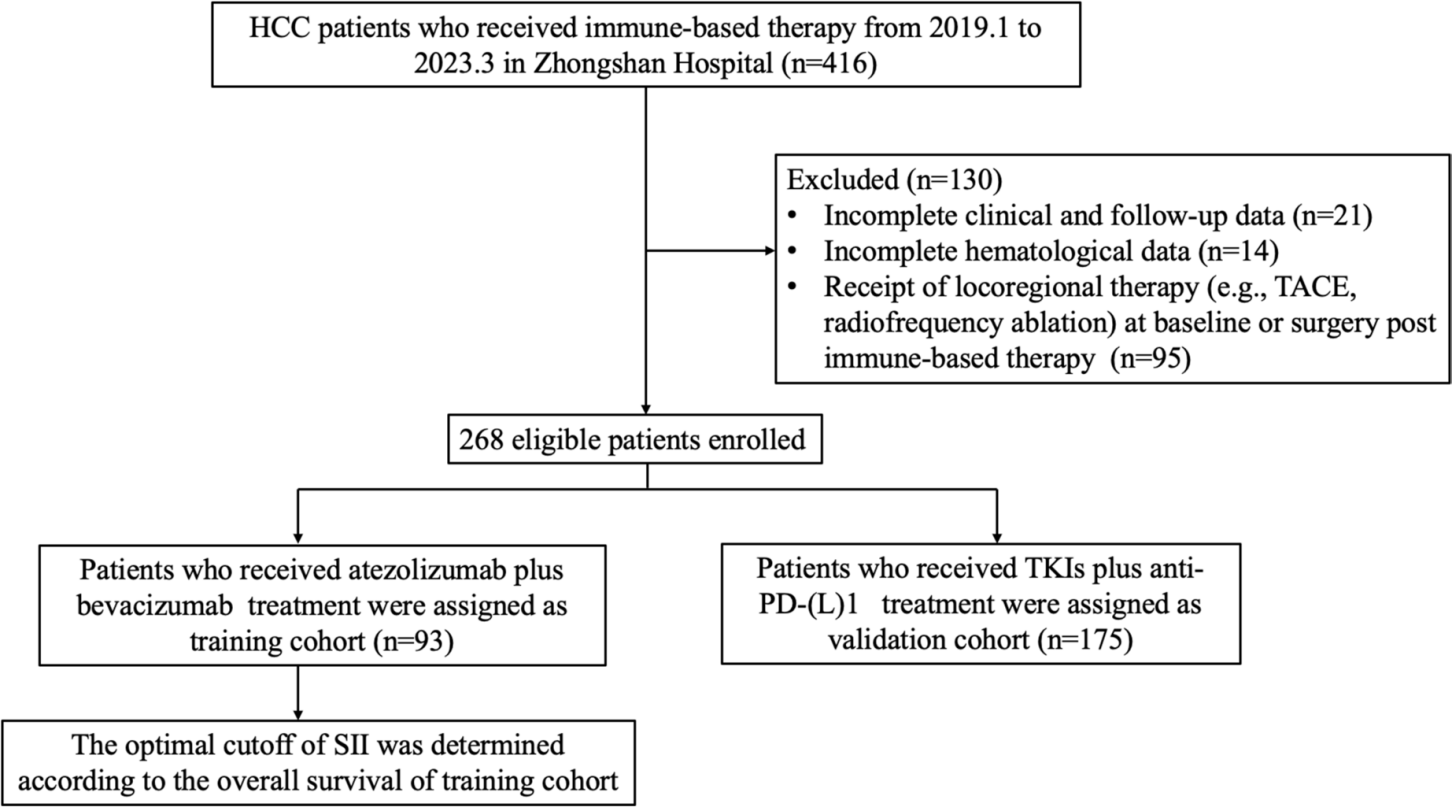
Flowchart of patient enrollment and the confirmation of optimal cutoff value for the SII

The study included 268 patients meeting the inclusion criteria, detailed in Fig. 1 and Table 1. Both cohorts, training and validation, were predominantly male (training: 90.32%, validation: 85.14%) and showed a high prevalence of advanced-stage HCC, as classified by BCLC (training: 94.62%, validation: 91.43%). A majority of patients tested positive for AFP and HBV, with compensated liver function. The training cohort had a lower incidence of extrahepatic metastasis (30.11% vs. 47.43%, *P* = 0.006) but a higher rate of HBsAg positivity (89.25% vs. 74.86%, *P* = 0.005) and macrovascular tumor thrombus (68.82% vs. 54.29%, *P* = 0.021) compared to the validation cohort. Additionally, the training group had a higher proportion of patients with lower PLR (76.34% vs. 64.00%, *P* = 0.039). No significant differences were noted in the distribution of patients based on SII and NLR between the cohorts.

**Table 1:** The clinicopathologic characteristics of patients in the training and validation cohorts

Variables	Training cohort		Validation cohort		
	*N* = 93	%	*N* = 175	%	*P*-value
Age(y)					
≤ 50	32	34.40%	53	30.29%	
> 50	61	65.60%	122	69.71%	0.490
Sex					
Female	9	9.68%	26	14.86%	
Male	84	90.32%	149	85.14%	0.231
Child–Pugh					
A	85	91.40%	156	89.14%	
B + C	8	8.60%	19	10.86%	0.559
HBsAg					
Negative	10	10.75%	44	25.14%	
Positive	83	89.25%	131	74.86%	0.005
ALT(U/L)					
≤ 50	73	78.49%	126	72.00%	
> 50	20	21.51%	49	28.00%	0.247
AFP (ng/mL)					
≤ 20	34	36.56%	52	29.71%	
> 20	59	63.44%	123	70.29%	0.253
Macrovascular tumor thrombus					
No	29	31.18%	80	45.71%	
Yes	64	68.82%	95	54.29%	0.021
Extrahepatic metastasis					
No	65	69.89%	92	52.57%	
Yes	28	30.11%	83	47.43%	0.006
BCLC					
A	5	5.38%	15	8.57%	
B + C	88	94.62%	160	91.43%	0.343
ECOG PS					
0	73	78.49%	118	67.43%	
1 + 2	20	21.51%	57	32.53%	0.057
NLR					
≤ 3	66	70.97%	108	61.71%	
> 3	27	29.03%	67	38.29%	0.131
PLR					
≤ 150	71	76.34%	112	64.00%	
> 150	22	23.66%	63	36.00%	0.039
SII					
≤ 752	72	77.42%	132	75.43%	
> 752	21	22.58%	43	24.57%	0.716

*Abbreviation: ALT* albumin, *AFP* alpha‐fetoprotein, *BCLC* Barcelona Clinic Liver Cancer, *NLR* neutrophil-to-lymphocyte ratio, *PLR* platelet-to-lymphocyte ratio, *SII* systemic immune-inflammation index

The training cohort showed a slightly higher objective response rate (ORR) of 30.1% compared to 29.7% in the validation cohort (Supplementary Figure. S2A). Median PFS was longer in the training group (7.37 months, 95% CI: 2.76 – 11.96) than in the validation cohort (6.50 months, 95% CI: 4.83 – 8.17), as depicted in Supplementary Figures S2B and S2C. Similarly, the training cohort exhibited a higher median OS of 17.7 months (95% CI: 14.7 – 29.2) compared to 16.77 months (95% CI: 13.18 – 20.35) in the validation group, as shown in Supplementary Figures S2D and S2E.

### Association between SII and clinicopathologic parameters

An optimal cutoff point for the SII of 752*10^9^ was identified in the training cohort using X-Tile analysis. Patients were subsequently categorized into two groups: SII ≤ 752*10^9^ or > 752*10^9^ for further analysis. Higher SII values (> 752*10^9^) correlated significantly with extrahepatic metastasis in the training cohort (*P* = 0.011) and with an increased likelihood of vascular tumor thrombus in the validation cohort (*P* = 0.019), as detailed in Table 2.

**Table 2:** The correlation between SII and clinicopathologic characteristics in training and validation cohorts

Variables	Training cohort(*n* = 93)					Validation cohort(*n* = 175)				
	SII ≤ 752	%	SII > 752	%	*P*-value	SII ≤ 752	%	SII > 752	%	*P*-value
Age(y)										
≤ 50	25	34.72%	7	33.33%		39	29.55%	14	32.56%	
> 50	47	65.28%	14	66.67%	0.906	93	70.45%	29	67.44%	0.709
Sex										
Female	7	9.72%	2	9.52%		19	14.39%	7	16.28%	
Male	65	90.28%	19	90.48%	0.978	113	85.61%	36	83.72%	0.763
Child–Pugh										
A	66	91.67%	19	90.48%		118	89.39%	38	88.37%	
B + C	6	8.33%	2	9.52%	0.864	14	10.61%	5	11.63%	0.852
HBsAg										
Negative	7	9.72%	3	14.29%		36	27.27%	8	18.60%	
Positive	65	90.28%	18	85.71%	0.553	96	72.73%	35	81.40%	0.255
ALT(U/L)										
≤ 50	55	76.39%	18	85.71%		92	69.70%	34	79.07%	
> 50	17	23.61%	3	14.29%	0.360	40	30.30%	9	20.93%	0.235
AFP (ng/mL)										
≤ 20	25	34.72%	9	42.86%		39	29.55%	14	32.56%	
> 20	47	65.28%	12	57.14%	0.496	93	70.45%	29	67.44%	0.709
Macrovascular tumor thrombus										
No	19	26.39%	10	47.62%		67	50.76%	13	30.23%	
Yes	53	73.61%	11	52.38%	0.065	65	49.24%	30	69.77%	0.019
Extrahepatic metastasis										
No	55	76.39%	10	47.62%		74	56.06%	18	41.86%	
Yes	17	23.61%	11	52.38%	0.011	58	43.94%	25	58.14%	0.105
BCLC										
A	3	4.17%	2	9.52%		14	25.76%	1	2.33%	
B + C	69	95.83%	19	90.48%	0.338	118	74.24%	42	97.67%	0.092
AEs										
No	57	79.17%	16	76.19%		106	80.31%	34	79.07%	
Yes	15	20.83%	5	23.81%	0.770	26	19.69%	9	20.93%	0.861

*Abbreviation: ALT* albumin, *AFP* alpha‐fetoprotein, *BCLC* Barcelona Clinic Liver Cancer, *NLR* neutrophil-to-lymphocyte ratio, *PLR* platelet-to-lymphocyte ratio, *SII* systemic immune-inflammation index, *AEs* the Immune-related adverse events

### Prognostic significance of SII in the training cohort

Building on previous findings regarding the prognostic value of the NLR in T + A treated HCC patients [23], this study focused on assessing the reliability of SII in a similar cohort from Zhongshan Hospital, Fudan University. Univariate analysis revealed SII as a significant prognostic factor for both PFS (HR = 2.296, 95% CI = 1.264 – 4.170, *P* = 0.006, Table 3) and OS (HR = 2.692, 95% CI = 1.395 – 5.196, *P* = 0.003, Table 4), outperforming other clinical factors. PLR and NLR were also significant for OS, as previously reported [21, 24]. Other factors such as age, gender, Child–Pugh score, alanine aminotransferase (ALT) level, α-fetoprotein (AFP), macrovascular tumor thrombus, extrahepatic metastasis, and CNLC stage showed limited prognostic impact for PFS or OS (Tables 3 and 4). Multivariate analysis confirmed SII as an independent prognostic factor for both PFS and OS.

**Table 3:** Univariate and multivariate cox regression analyses of the SII with clinicopathologic characteristics for PFS [training cohort (*n* = 93) and validation cohort (*n* = 175)]

Variable	Category	Univariate analysis		Multivariate analysis	
		HR (95% CI)	P-value	HR (95% CI)	*P*-value
Training cohort					
Age(y)	> 50 vs. ≤ 50	1.524 (0.829—2.803)	0.175		
Sex	Male vs. Female	1.402 (0.433—4.543)	0.573		
Child–Pugh	B vs. A	1.155 (0.406—3.281)	0.787		
HBsAg	Positive vs. Negative	1.440 (0.444—4.671)	0.544		
ALT(U/L)	> 50 vs. ≤ 50	1.626 (0.796—3.320)	0.182		
AFP (ng/mL)	> 20 vs. ≤ 20	1.657 (0.878—3.129)	0.119		
Macrovascular tumor thrombus	Yes vs. No	1.604 (0.810—3.175)	0.175		
Extrahepatic metastasis	Yes vs. No	1.093 (0.584—2.045)	0.782		
BCLC	B + C vs. A	1.071 (0.205—5.605)	0.935		
NLR	> 3 vs. ≤ 3	1.248 (0.665—2.345)	0.490		
PLR	> 150 vs. ≤ 150	1.780 (0.951—3.334)	0.072	1.252 (0.611—2.563)	0.539
SII	> 752 vs. ≤ 752	2.296 (1.264—4.170)	0.006	2.084 (1.059—4.100)	0.033
Validation cohort					
Age(y)	> 50 vs. ≤ 50	1.229 (0.779—1.938)	0.376		
Sex	Male vs. Female	0.906 (0.479—1.715)	0.763		
Child–Pugh	B vs. A	1.050 (0.506—2.180)	0.896		
HBsAg	Positive vs. Negative	1.124 (0.672—1.879)	0.657		
ALT(U/L)	> 50 vs. ≤ 50	1.594 (1.003—2.536)	0.049	1.672 (1.047—2.670)	0.031
AFP (ng/mL)	> 20 vs. ≤ 20	1.125 (0.695—1.819)	0.632		
Macrovascular tumor thrombus	Yes vs. No	1.286 (0.825—2.005)	0.267		
Extrahepatic metastasis	Yes vs. No	1.379 (0.898—2.119)	0.142		
BCLC	B + C vs. A	1.512 (0.558—4.103)	0.416		
NLR	> 3 vs. ≤ 3	1.338 (0.870 – 2.059)	0.185		
PLR	> 150 vs. ≤ 150	1.466 (0.949—2.264)	0.085	0.804 (0.455—1.421)	0.454
SII	> 752 vs. ≤ 752	2.546 (1.614—4.015)	< 0.001	2.602 (1.485—4.561)	< 0.001

*Abbreviation: albumin* AFP, alpha‐fetoprotein, *BCLC* Barcelona Clinic Liver Cancer, *NLR *neutrophil-to-lymphocyte ratio, *PLR* platelet-to-lymphocyte ratio, *SII* systemic immune-inflammation index

**Table 4:** Univariate and multivariate cox regression analyses of the SII with clinicopathologic characteristics for OS [training cohort (*n* = 93) and validation cohort (*n* = 175)]

Variable	Category	Univariate analysis		Multivariate analysis	
		HR (95% CI)	*P*-value	HR (95% CI)	*P*-value
Training cohort					
Age(y)	> 50 vs. ≤ 50	1.214 (0.626—2.353)	0.566		
Sex	Male vs. Female	1.445 (0.499—4.184)	0.497		
Child–Pugh	B vs. A	1.226 (0.374—4.022)	0.737		
HBsAg	Positive vs. Negative	1.256 (0.383—4.124)	0.706		
ALT(U/L)	> 50 vs. ≤ 50	1.224 (0.533—2.810)	0.633		
AFP (ng/mL)	> 20 vs. ≤ 20	1.256 (0.383—4.124)	0.882		
Macrovascular tumor thrombus	Yes vs. No	0.978 (0.492—1.948)	0.951		
Extrahepatic metastasis	Yes vs. No	0.978 (0.492—1.948)	0.059	1.210 (0.556—2.633)	0.630
BCLC	B + C vs. A	1.412 (0.245—8.126)	0.699		
NLR	> 3 vs. ≤ 3	2.126 (1.120—4.038)	0.021	1.171 (0.440—3.120)	0.752
PLR	> 150 vs. ≤ 150	2.098 (1.081—4.072)	0.029	2.098 (1.081—4.072)	0.669
SII	> 752 vs. ≤ 752	2.692 (1.395—5.196)	0.003	2.330 (1.094—4.962)	0.028
Validation cohort					
Age(y)	> 50 vs. ≤ 50	0.909 (0.617—1.338)	0.628		
Sex	Male vs. Female	1.164 (0.675—2.005)	0.585		
Child–Pugh	B vs. A	1.405 (0.712—2.769)	0.327		
HBsAg	Positive vs. Negative	1.339 (0.883—2.031)	0.170		
ALT(U/L)	> 50 vs. ≤ 50	1.423 (0.970—2.088)	0.071	1.421 (0.963—2.098)	0.077
AFP (ng/mL)	> 20 vs. ≤ 20	1.033 (0.701—1.523)	0.869		
Macrovascular tumor thrombus	Yes vs. No	1.343 (0.940—1.919)	0.106		
Extrahepatic metastasis	Yes vs. No	0.972 (0.683—1.383)	0.875		
BCLC	B + C vs. A	1.284 (0.454—3.633)	0.637		
NLR	> 3 vs. ≤ 3	1.682 (1.175—2.407)	0.004	1.396 (0.943—2.066)	0.096
PLR	> 150 vs. ≤ 150	1.733 (1.212—2.477)	0.003	1.249 (0.821—1.899)	0.298
SII	> 752 vs. ≤ 752	2.148 (1.456—3.168)	< 0.001	1.664 (1.051—2.634)	0.030

*Abbreviation: ALT* albumin, *AFP* alpha‐fetoprotein, *BCLC* Barcelona Clinic Liver Cancer, *NLR* neutrophil-to-lymphocyte ratio, *PLR* platelet-to-lymphocyte ratio, *SII* systemic immune-inflammation index

**Fig. 2:**
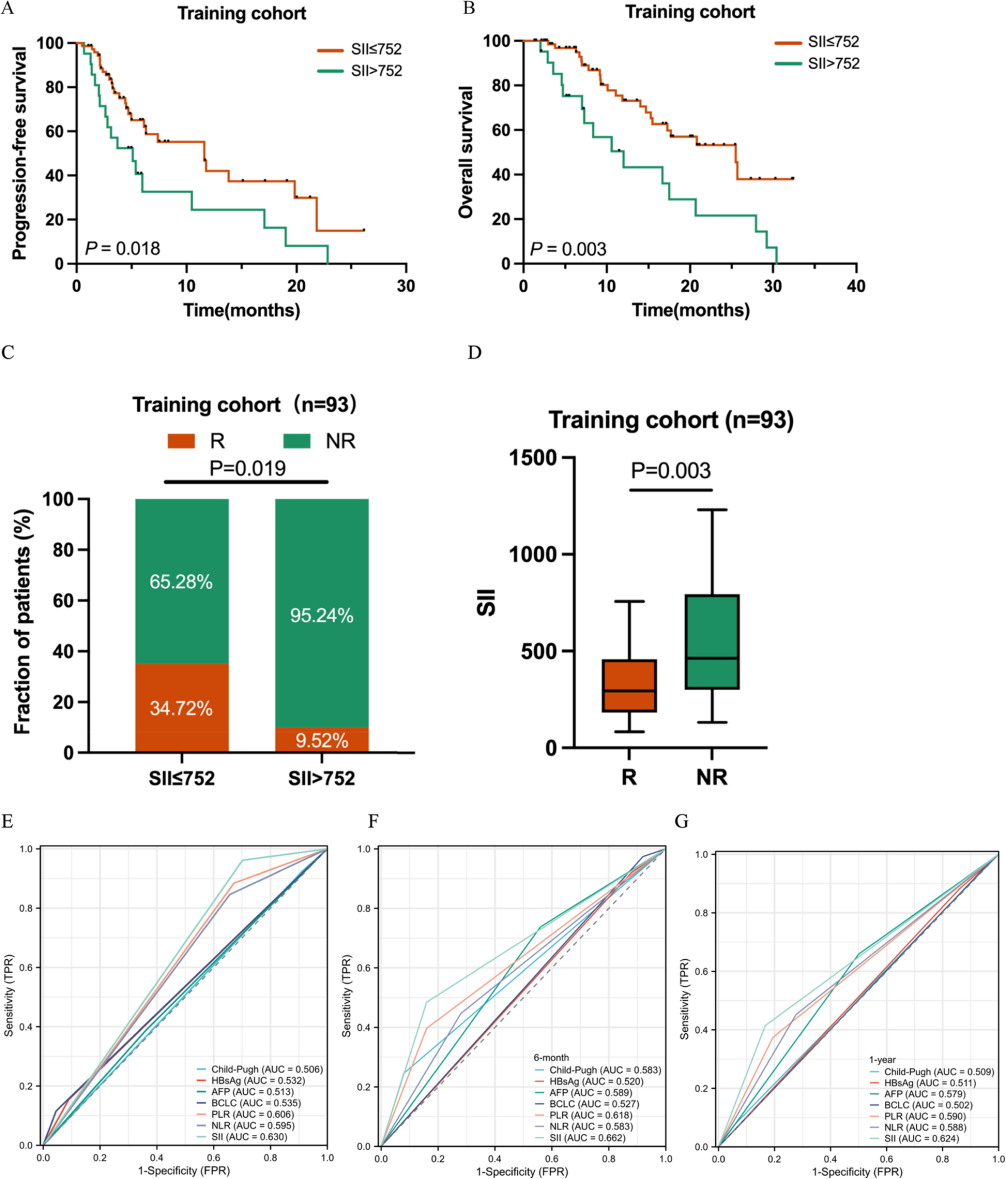
The Kaplan–Meier analysis of PFS (**A**) and OS (**B**) for the SII in the training cohort. Comparison of ORR for the SII in the training cohort (**C**). The comparison of SII in responders and non-responders in the training cohort (**D**). Predictive ability of the SII was compared with other clinical parameters by ROC curves in the training cohorts (**E**–**G**)

Kaplan–Meier analysis demonstrated that lower SII was associated with longer PFS and OS (Fig. 2A - B). Patients with SII ≤ 752*10^9^ had a median PFS of 11.6 months and OS of 25.5 months, whereas those with SII > 752*10^9^ had 5.1 months and 12 months, respectively. Further analysis for PLR and NLR indicated that lower PLR correlated with longer PFS and OS (Supplementary Figure. S3A—B), while lower NLR only showed a significant association with OS (Supplementary Figure. S3C—D). Patients with lower SII had better ORR (34.72% vs. 9.52%; *P* = 0.019; Fig. 2C; Supplementary Table S1). NLR and PLR had limited predictive ability for ORR (Supplementary Figure. S3E—F). Additionally, responders tended to have lower SII scores than non-responders (337.0 vs. 613.3, *P* < 0.001, Fig. 2D). The area under the curve (AUC) values for SII, NLR, and PLR in predicting ORR, OS, and PFS suggested that SII was the strongest factor among them (Fig. 2E - G). Delong test showed that the AUC of SII have marginally significant over PLR and NLR in predicting PFS (*P* = 0.238, *P* = 0.133, respectively). Although there were no significant statistical differences in ORR (*P* = 0.665, *P* = 0.407, respectively) and OS (*P* = 0.774, *P* = 0.280, respectively), SII still took the lead. We also analyzed time-dependent AUC and C-index to make further confirmation (Supplementary Figure. S3G—J).

### Validation of the SII in an independent cohort

**Fig. 3:**
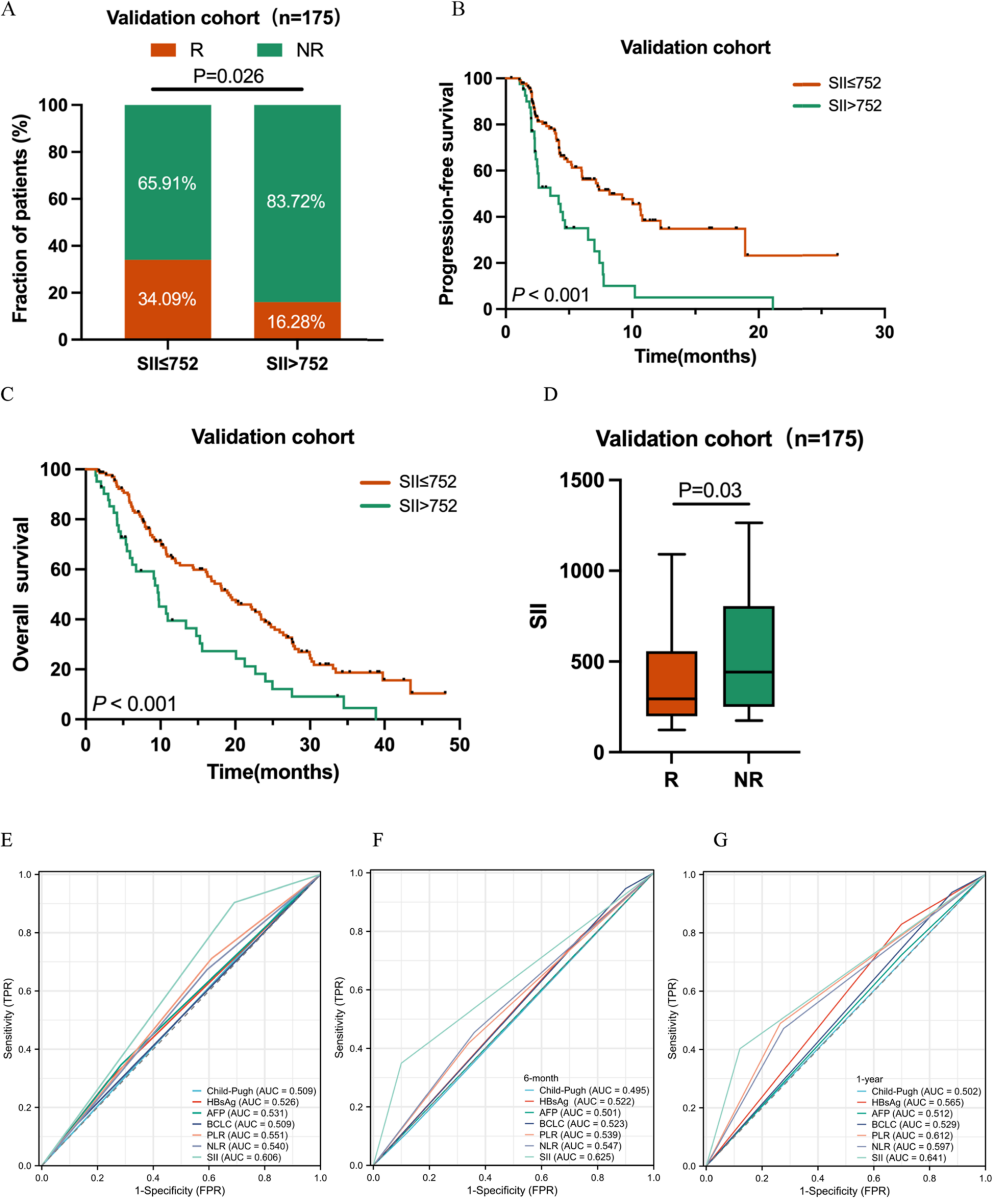
Comparison of ORR for the SII in the validation cohort (**A**). The Kaplan–Meier analysis of PFS (**B**) and OS (**C**) for the SII in the validation cohorts. The comparison of SII in responders and non-responders in the validation cohort (**D**). Predictive ability of the SII was compared with other clinical parameters by ROC curves in the validation cohorts (**E**–**G**)

The prognostic relevance of SII was further validated in an independent cohort of 175 patients treated with TKIs plus PD-(L)1 inhibitors. The validation cohort’s findings were consistent with the initial training cohort, confirming the strong association of SII with PFS and OS in both univariate and multivariate analyses (Tables 3 and 4). Patients with SII lower than 752*10^9^ showed a higher response rate and benefited more from immune-based therapy, evidenced by prolonged PFS and OS (Fig. 3A - C). Responders were characterized by significantly lower SII scores compared to non-responders (427.8 vs. 649.4, *P* = 0.005, Fig. 3D). The predictive function of PLR for PFS was not observed in this cohort (Supplementary Figure. S4). The AUC values for SII in predicting ORR, PFS, and OS were superior to other clinical indices (Fig. 3E - G). Delong test revealed that as for predicting PFS, SII had slightly significance over PLR (*P* = 0.175) and NLR (*P* = 0.107). The time-dependent AUC also showed the similar results and the C-index proved the stability of SII’s predictive function (Supplementary Figure. S4G—J).

### The prognostic significance of SII in HCC patients with negative AFP subgroups

**Fig. 4:**
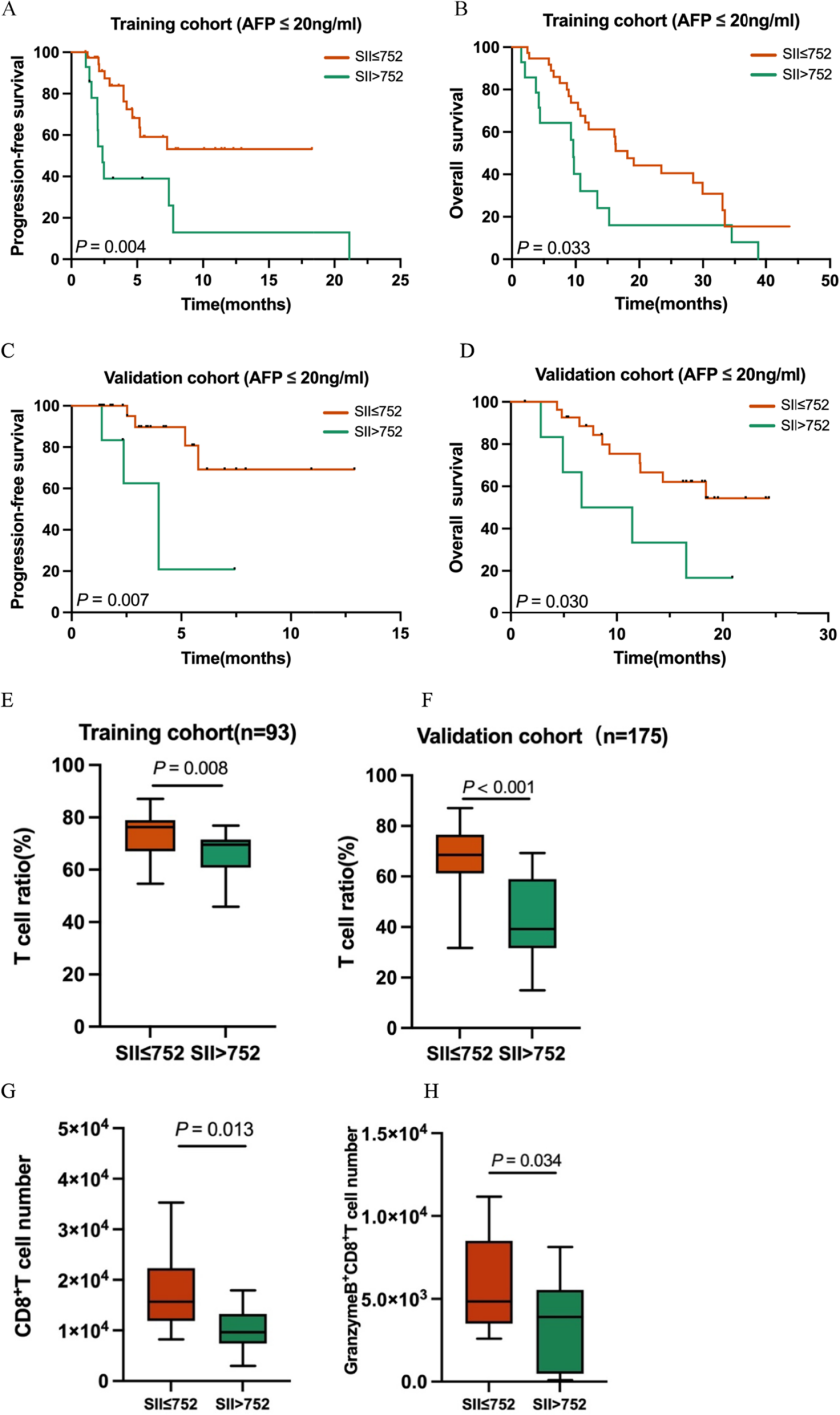
The prognostic significance of the SII in patients with HCC in negative AFP subgroups. The Kaplan–Meier analysis of PFS and OS for the SII in AFP 20 ≤ ng/mL (**A**—**D**) in the training and validation cohorts. Patients in SII ≤ 752 subgroup had higher T, CD8^+^T and GranzymeB^+^CD8^+^T cell ratio in the training and validation cohort (**E**–**H**)

Further investigation into the prognostic value of SII in AFP-negative HCC patients revealed that lower SII scores in the training cohort significantly improved PFS and OS (Fig. 4A - B). The validation cohort showed similar findings, confirming the prognostic relevance of SII for PFS and OS in AFP-negative patients (Fig. 4C - D). These results highlight SII’s potential as a valuable biomarker in this specific patient subgroup.

### Patients with lower SII scores tend to have higher effective T cell eatio in peripheral blood

Exploring the relationship between SII scores and immune response, the study focused on lymphocyte composition, particularly T cells, given their established significance in cancer immune-based therapy [25]. Patients with lower SII scores exhibited higher T cell ratios in their lymphocytes in both cohorts (*P* = 0.008, *P* < 0.001, respectively, Fig. 4E - F). According to the flow cytometry results in our previous study [26], we matched 33 patients in TKIs plus PD-(L)1 antibody group. Results showed that patients in lower SII group tended to have higher CD8^+^T cell and GranzymeB^+^CD8^+^T cell number in peripheral blood (*P* = 0.013, *P* = 0.034, respectively, Fig. 4G - H). These findings underscore the potential influence of SII on immune functionality, especially considering the crucial role of T cells in mediating anti-tumor effects.

## Discussion

The remarkable progress in immune-based therapy for HCC, particularly with the emergence of combination therapies such as atezolizumab and bevacizumab, represents a substantial advancement in the treatment of advanced-stage HCC [27]. Despite this progress, challenges remain, most notably the suboptimal response rate to these therapies. This underscores the urgent need for effective predictive biomarkers [28], making our study’s focus on the SII as a prognostic tool both timely and crucial.

In our analysis, we underscore the well-established link between inflammation and anti-tumor immune response [29]. The SII, comprising platelets, neutrophils, and lymphocytes, serves as a potential indicator of the balance between inflammation and immune response. Our findings reveal that an elevated SII, often indicative of abnormal inflammatory states and weaker immune responses, is associated with poorer outcomes in immune-based therapy. This is particularly relevant since inflammation plays a pivotal role in tumor progression [30], and components like platelets and neutrophils are known to promote tumor growth and immune evasion, whereas a reduced lymphocyte count usually indicates a weakened immune response [31–33].

AFP is the most widely used biomarker for HCC, its predictive ability is limited in AFP-negative patients [34]. This limitation underscores a significant gap in current diagnostic and prognostic approaches [35]. Our study addresses this by demonstrating that SII retains its prognostic value even in AFP-negative patients. We observed distinct differences in OS and PFS among patients with varying SII scores in both training and validation cohorts. These observations reinforce the potential of SII as a valuable prognostic marker in this challenging subgroup, opening new possibilities for patient stratification and management.

Further investigating the mechanistic underpinnings of SII scores, we found that lower SII scores correlate with a higher T cell ratio in lymphocytes. Based on the previous flow cytometry results, we deeply looked into the detailed composition of T cells and gladly found that patients with lower SII had higher CD8^+^T cell and GranzymeB^+^CD8^+^T cell number in peripheral blood. This correlation is significant, considering the vital role of effective T cells in immune-based therapy and anti-tumor responses. A higher number of effective T cells suggests a more potent anti-tumor response. These finding sheds light on how SII may influence patient responses to immune-based therapy and suggests further research, particularly focusing on the subtypes and functional states of T cells [26].

Our study is pioneering in demonstrating the prognostic value of baseline SII in HCC patients undergoing treatment with the atezolizumab-bevacizumab combination, a frontline therapy for advanced-stage HCC. We also validated SII’s functionality in patients treated with TKIs plus anti-PD-(L)1, suggesting its broad applicability in predicting outcomes and guiding treatment decisions, especially in AFP-negative patients who traditionally lack effective clinical indicators [36]. Given that SII is a further development based on NLR and PLR, it did show an edge over these two classic biomarkers in the prediction of both ORR, OS and PFS, showing its greater potential. Compared with other predictive models like MRI-based Radiomic, SII is easier to obtain and time-saving, which is also more economical. The standard of blood routine is more stable compared with current MRI image acquisition parameters [37, 38]. These findings could significantly refine clinical practice, allowing for a more nuanced approach to HCC management.

While our findings are promising, we acknowledge the limitations of our study, such as its single-center nature and the limited patient sample. To generalize our findings, broader validation across diverse patient populations is essential. Also, prospective data will also be more effective to verify the accuracy of SII, which could be realized in the future prospective studies. Moreover, the complete mechanism behind SII warrants further exploration and validation. Understanding the complex interplay between different components of the immune system and their impact on immune-based therapy outcomes remains a key area for future research.

Additionally, future studies should examine the dynamic changes in SII during treatment to assess their potential predictive value. Investigating these longitudinal variations could offer deeper insights into immune response dynamics under therapeutic pressure and their implications for patient prognosis.

In conclusion, our study underscores the potential of SII as a predictive biomarker for HCC patients undergoing immune-based therapy. The simplicity, cost-effectiveness, and wide-applicability of the SII position it as a promising tool for assessing HCC prognosis in clinical practice. With further validation and exploration, SII could significantly contribute to personalized medicine, aiding in the selection of patients most likely to benefit from specific immune-based therapies.

## Supplementary Information

Supplementary Material 1: Supplementary Fig. 1. The optimal cutoff value for the SII was selected by X-tile 3.6.1 software (Yale University, New Haven, CT, USA). Supplementary Fig. 2. The ORR in training and validation cohort (A). The Kaplan–Meier analysis of PFS and OS in the training and validation cohorts (B—E). Supplementary Fig. 3. The Kaplan–Meier analysis of PFS (A) and OS (B) for the PLR in the training cohort. The Kaplan–Meier analysis of PFS (C) and OS (D) for the NLR in the training cohort. Comparison of ORR for the NLR and PLR in the training cohort (E–F). Time-dependent AUC and the C-index of OS and PFS for SII in the training cohort (G—J). Supplementary Fig. 4. Comparison of ORR for the NLR and PLR in the validation cohort (A-B). The Kaplan–Meier analysis of PFS and OS for the PLR and NLR in the validation cohort (C—F). Time-dependent AUC and the C-index of OS and PFS for SII in the validation cohort (G—J).

## Data Availability

No datasets were generated or analysed during the current study.
