# Supplementary material for: The prognostic value of systemic immune-inflammation index in patients with unresectable hepatocellular carcinoma treated with immune-based therapy
Source: Biomark Res. 2025 Jan 14;13:10. doi: 10.1186/s40364-024-00722-6 (PMC11730499; doi:10.1186/s40364-024-00722-6)
Supplement: Supplementary file 1 — Supplementary Material 1: Supplementary Fig. 1. The optimal cutoff value for the SII was selected by X-tile 3.6.1 software (Yale University, New Haven, CT, USA). Supplementary Fig. 2. The ORR in training and validation cohort (A). The Kaplan–Meier analysis of PFS and OS in the training and validation cohorts (B—E). Supplementary Fig. 3. The Kaplan–Meier analysis of PFS (A) and OS (B) for the PLR in the training cohort. The Kaplan–Meier analysis of PFS (C) and OS (D) for the NLR in the training cohort. Comparison of ORR for the NLR and PLR in the training cohort (E–F). Time-dependent AUC and the C-index of OS and PFS for SII in the training cohort (G—J). Supplementary Fig. 4. Comparison of ORR for the NLR and PLR in the validation cohort (A-B). The Kaplan–Meier analysis of PFS and OS for the PLR and NLR in the validation cohort (C—F). Time-dependent AUC and the C-index of OS and PFS for SII in the validation cohort (G—J). [file 40364_2024_722_MOESM1_ESM.docx]

**Supplementary figures**

**Supplementary Figure S1**


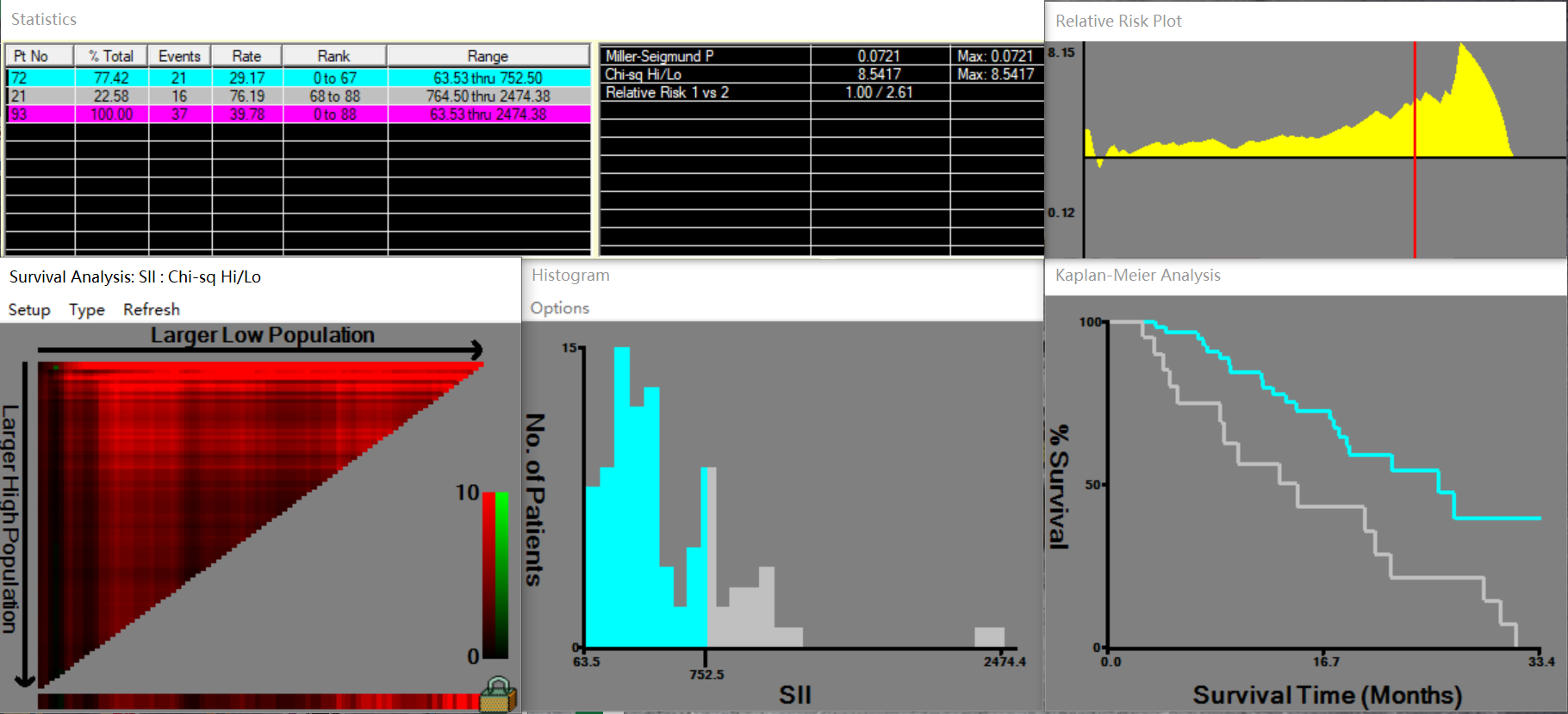


**Supplementary Figure S1.** The optimal cutoff value for the SII was selected by X-tile 3.6.1 software (Yale University, New Haven, CT, USA).

**Supplementary Figure S2**

A


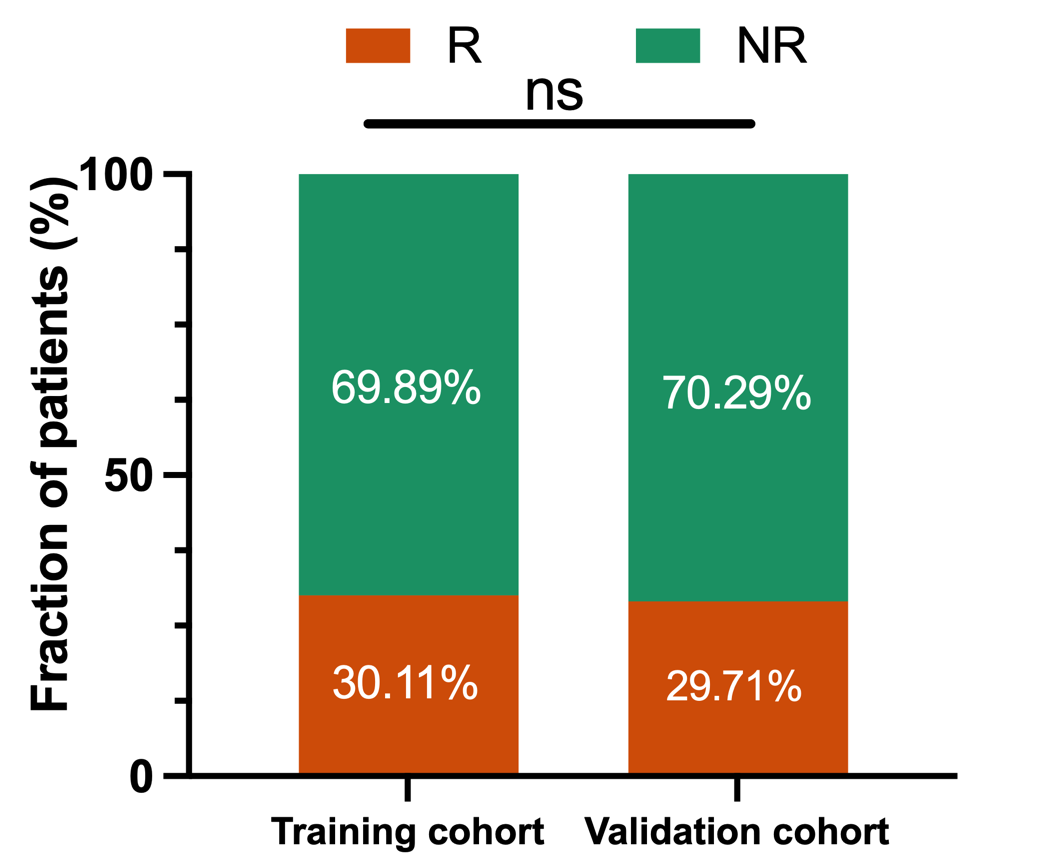


B C


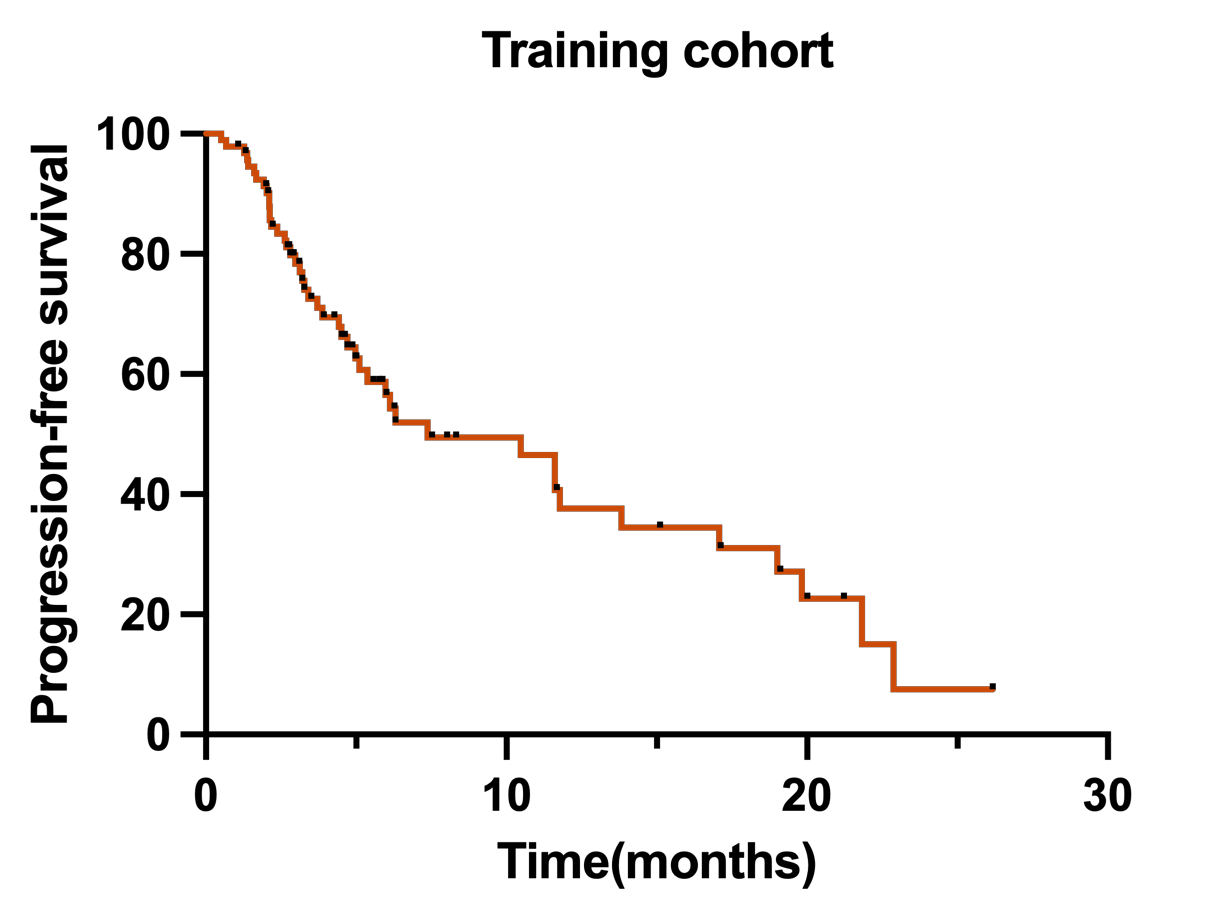

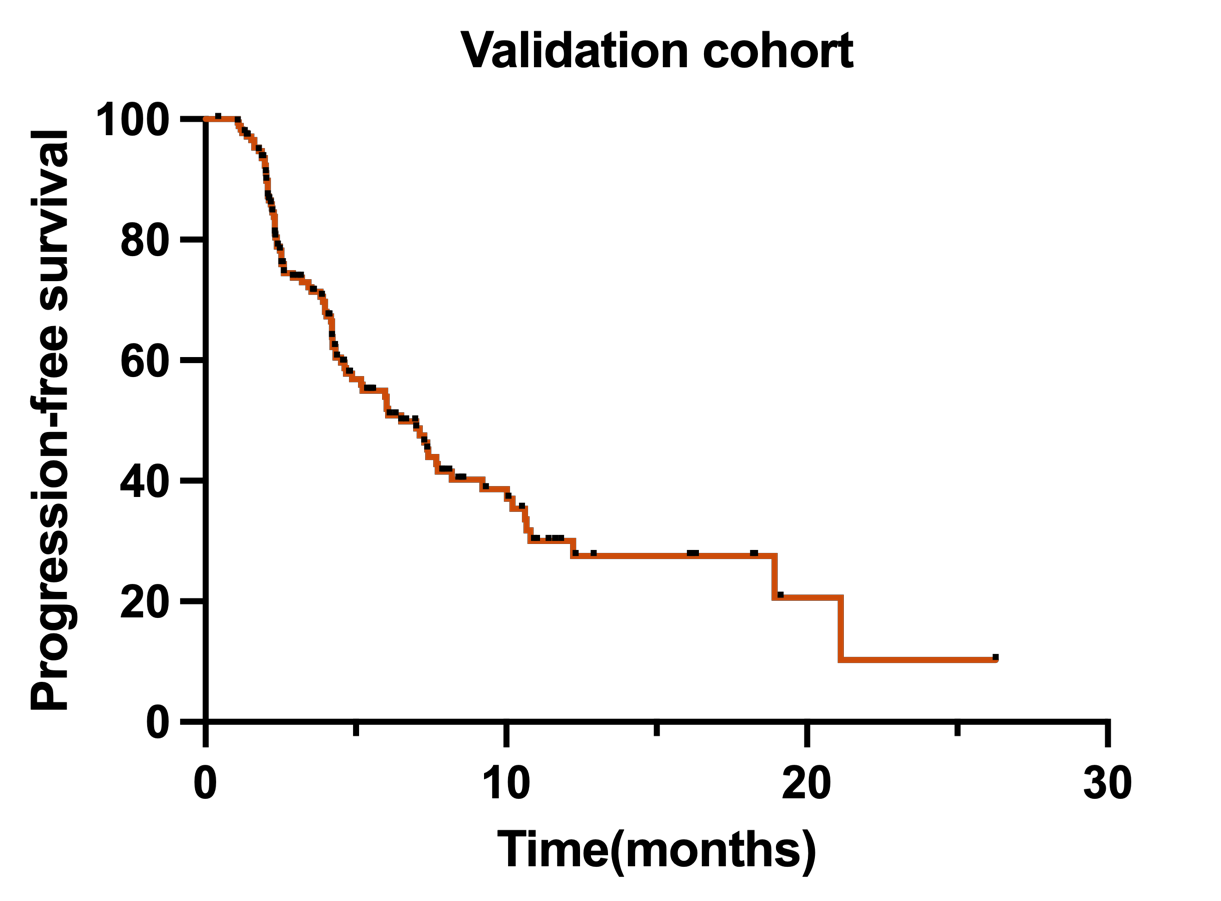


D E


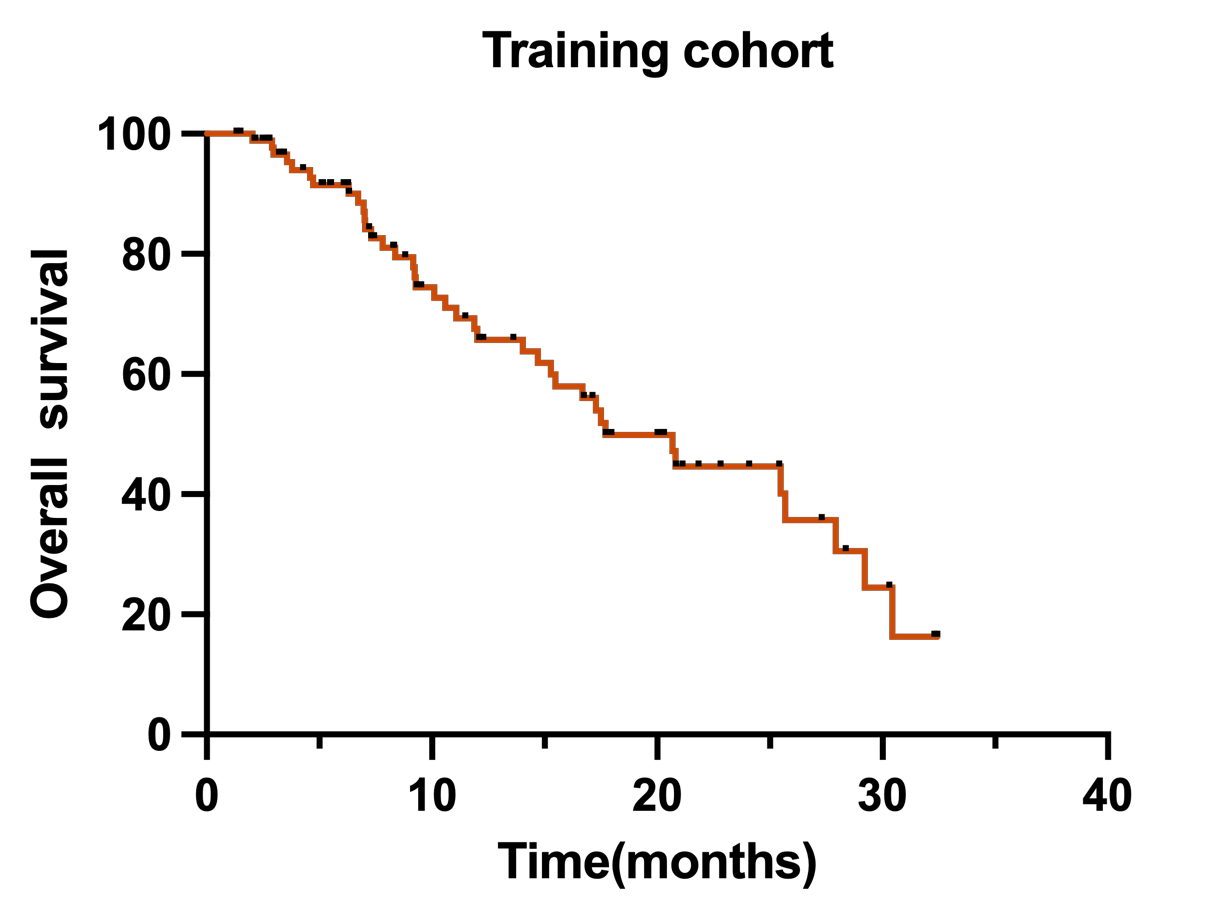

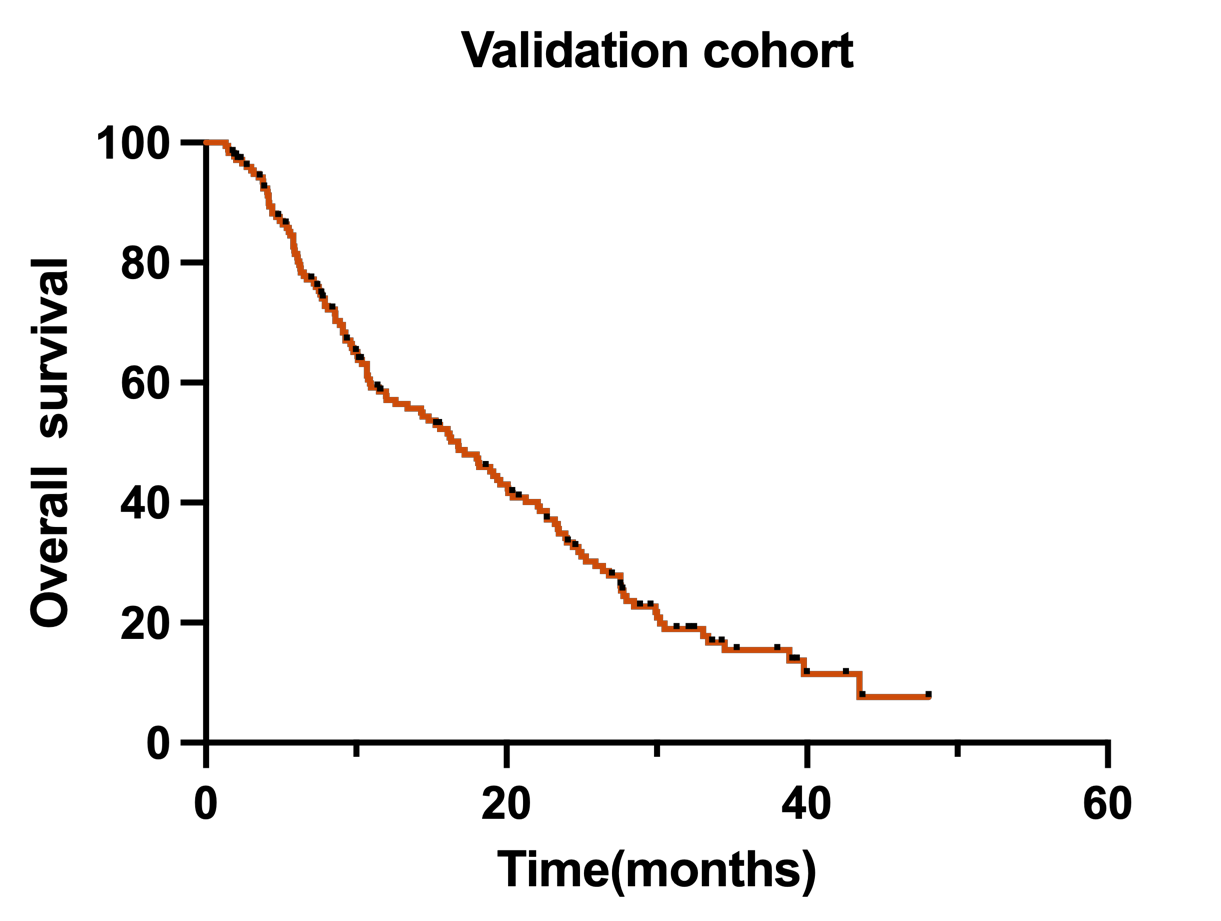


**Supplementary Figure S2.** The ORR in training and validation cohort (A). The Kaplan–Meier analysis of PFS and OS in the training and validation cohorts (B - E).

**Supplementary Figure S3**

A B


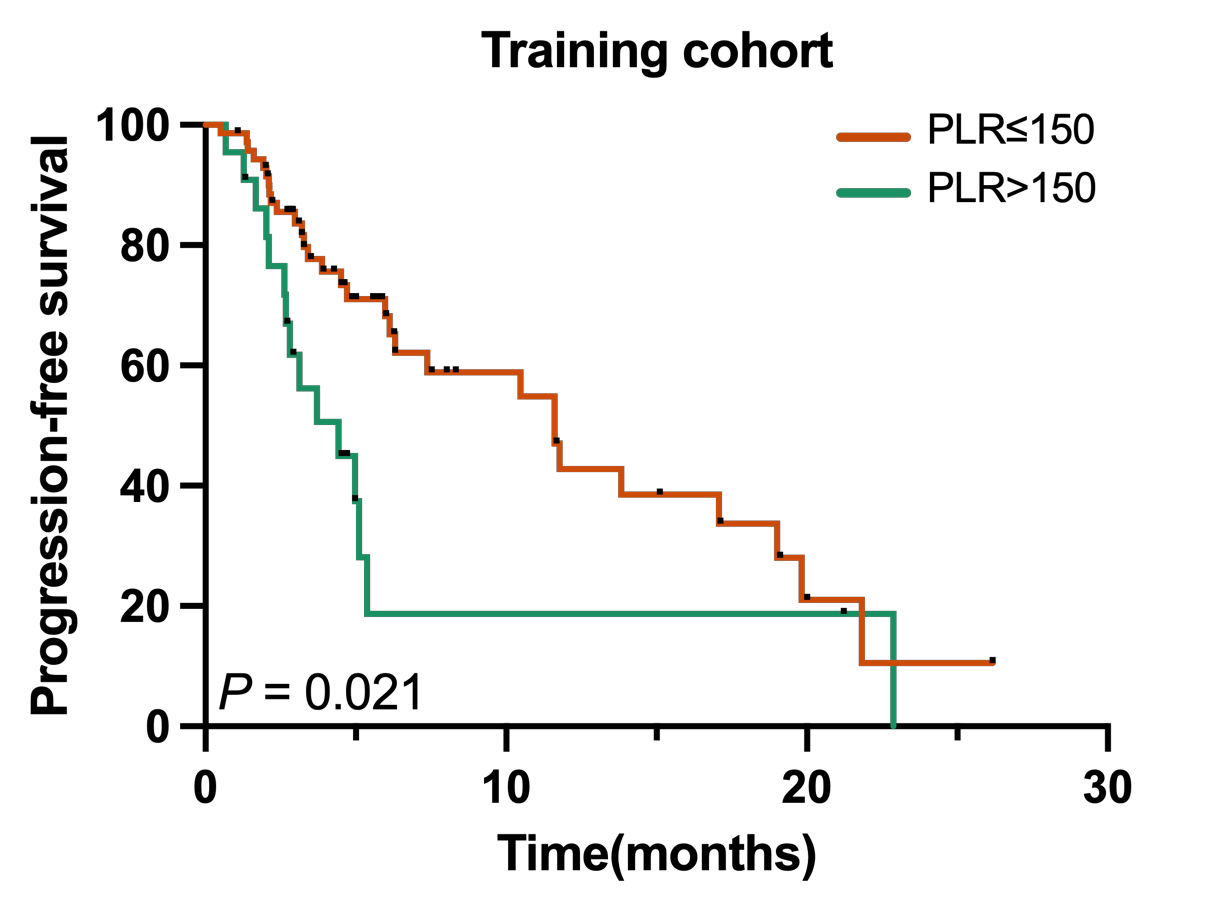

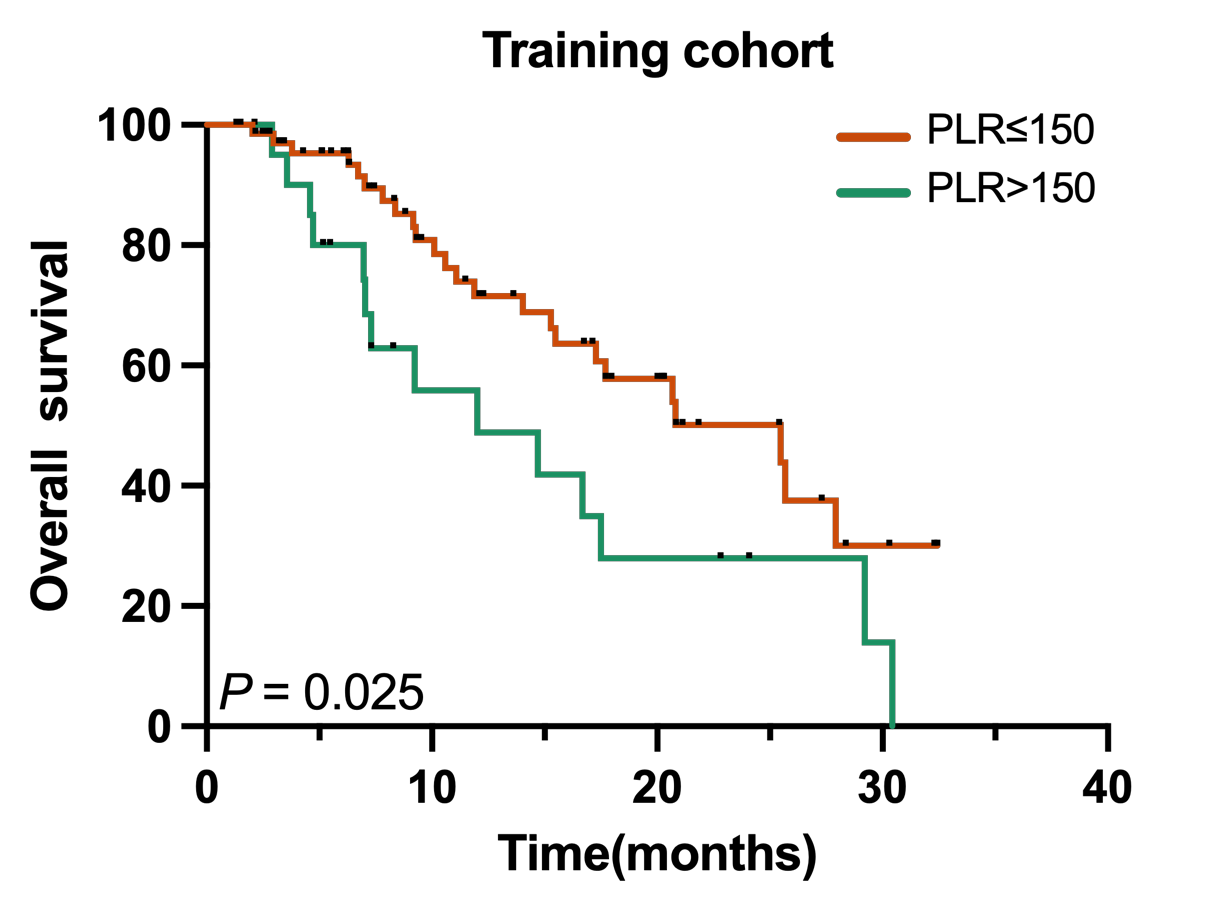


C D


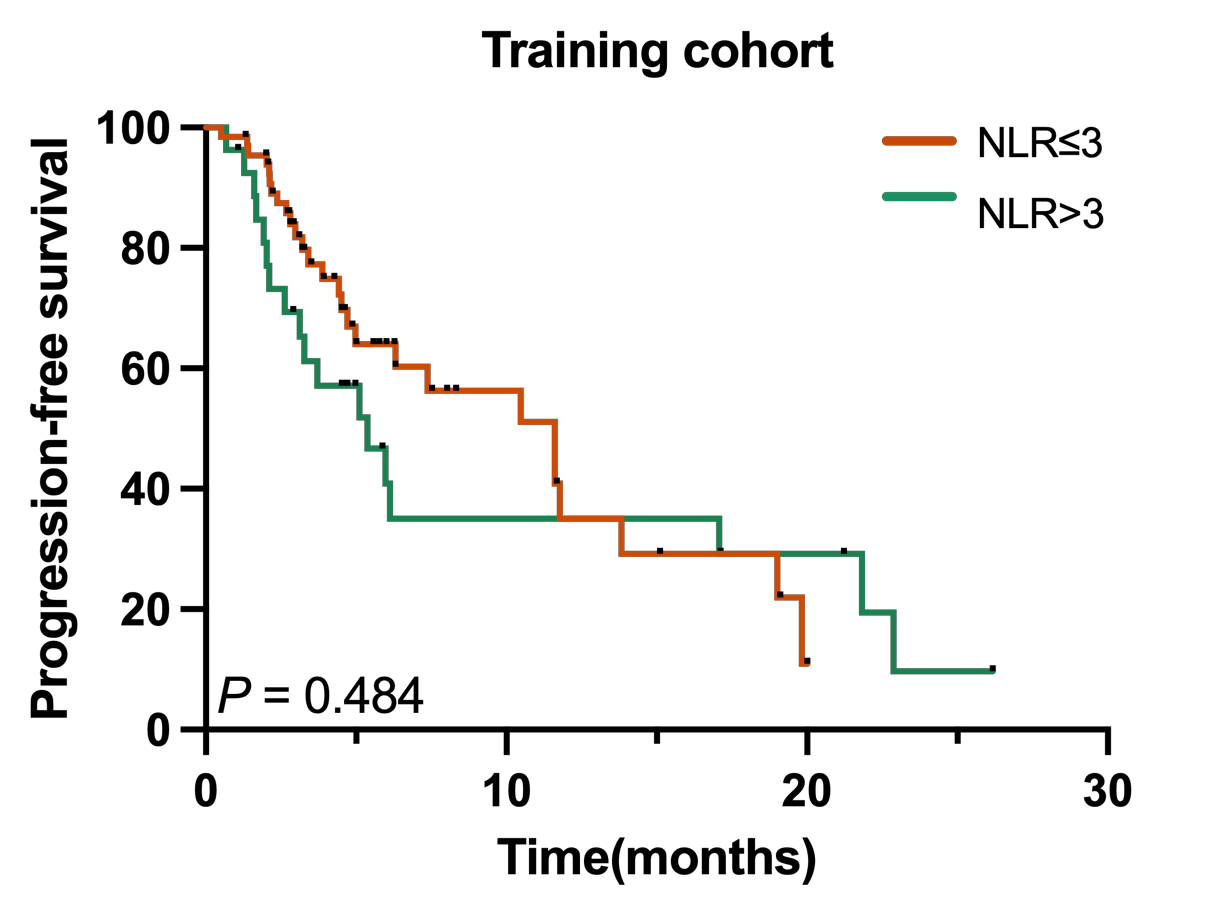

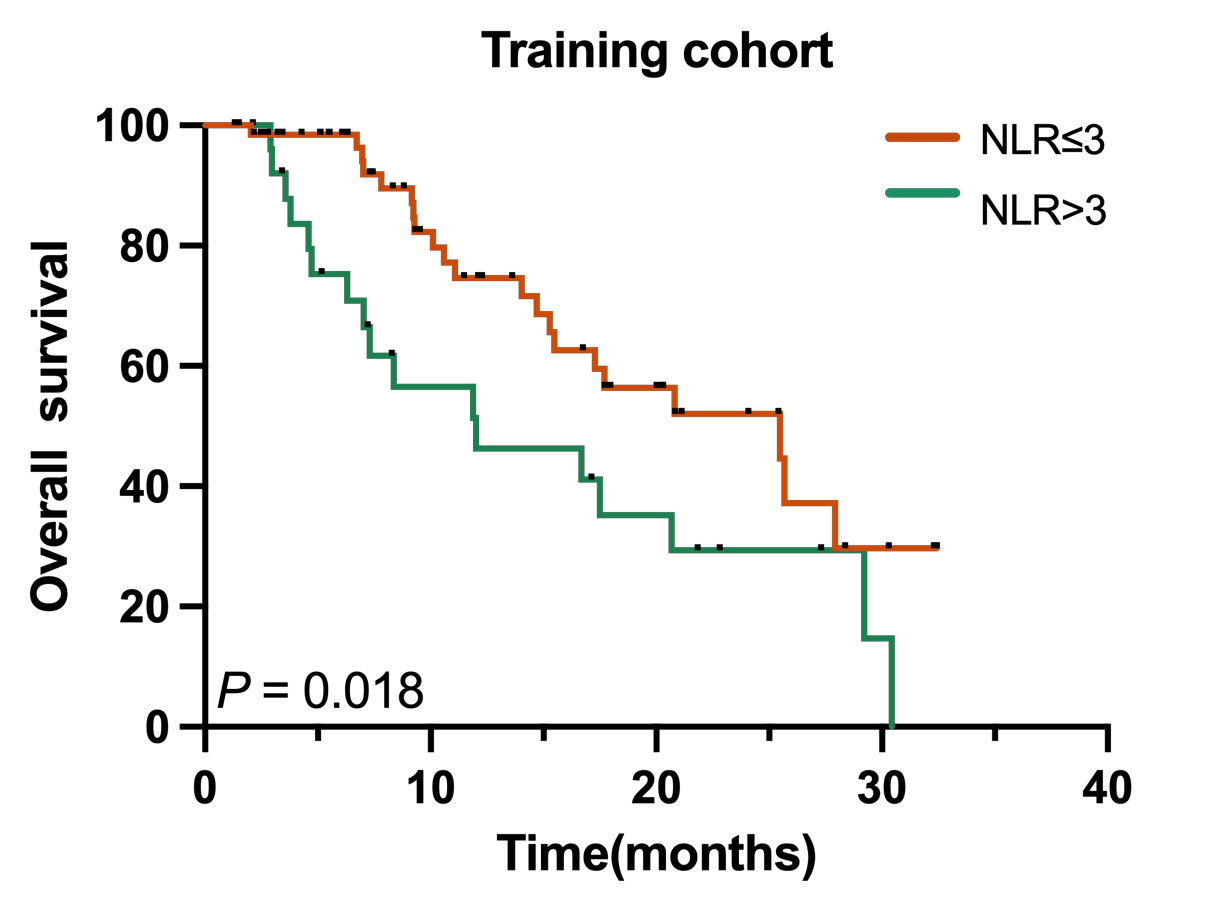


E F


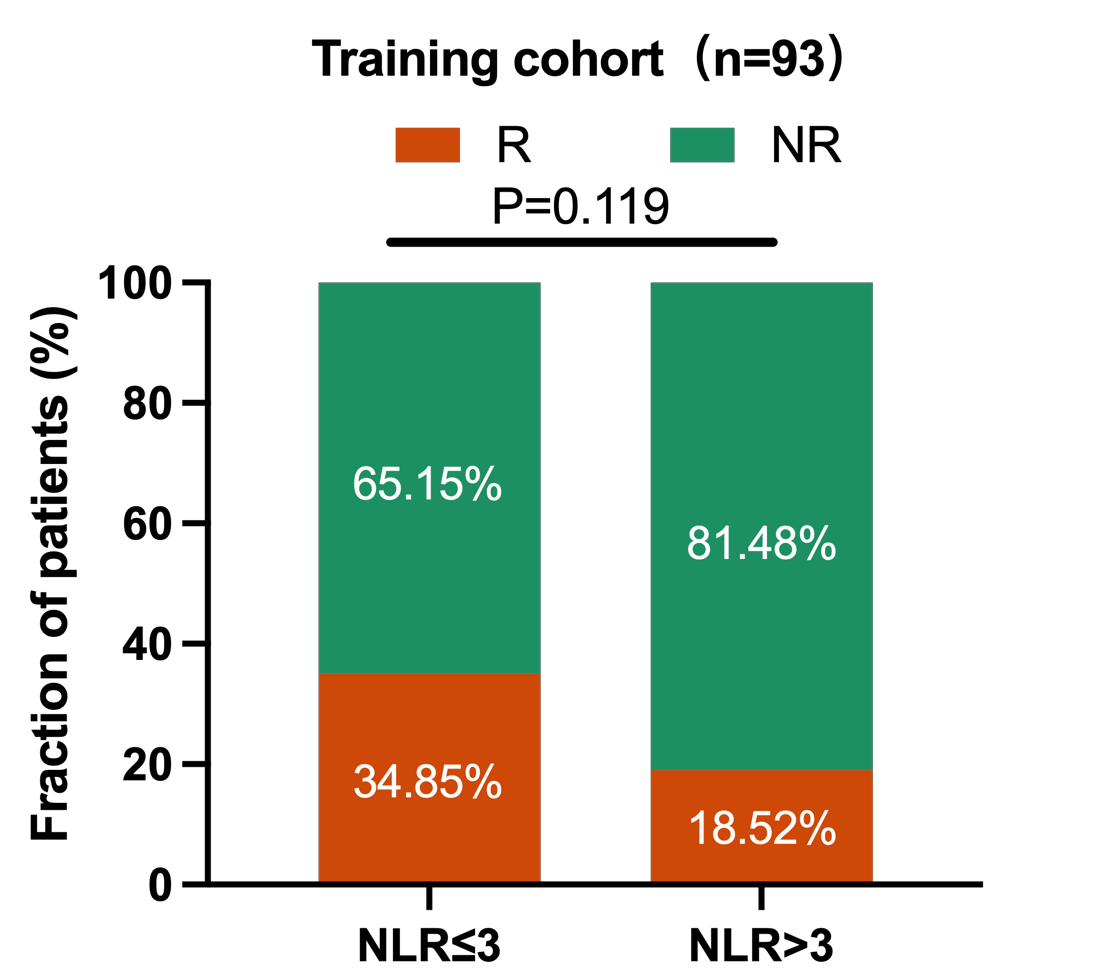

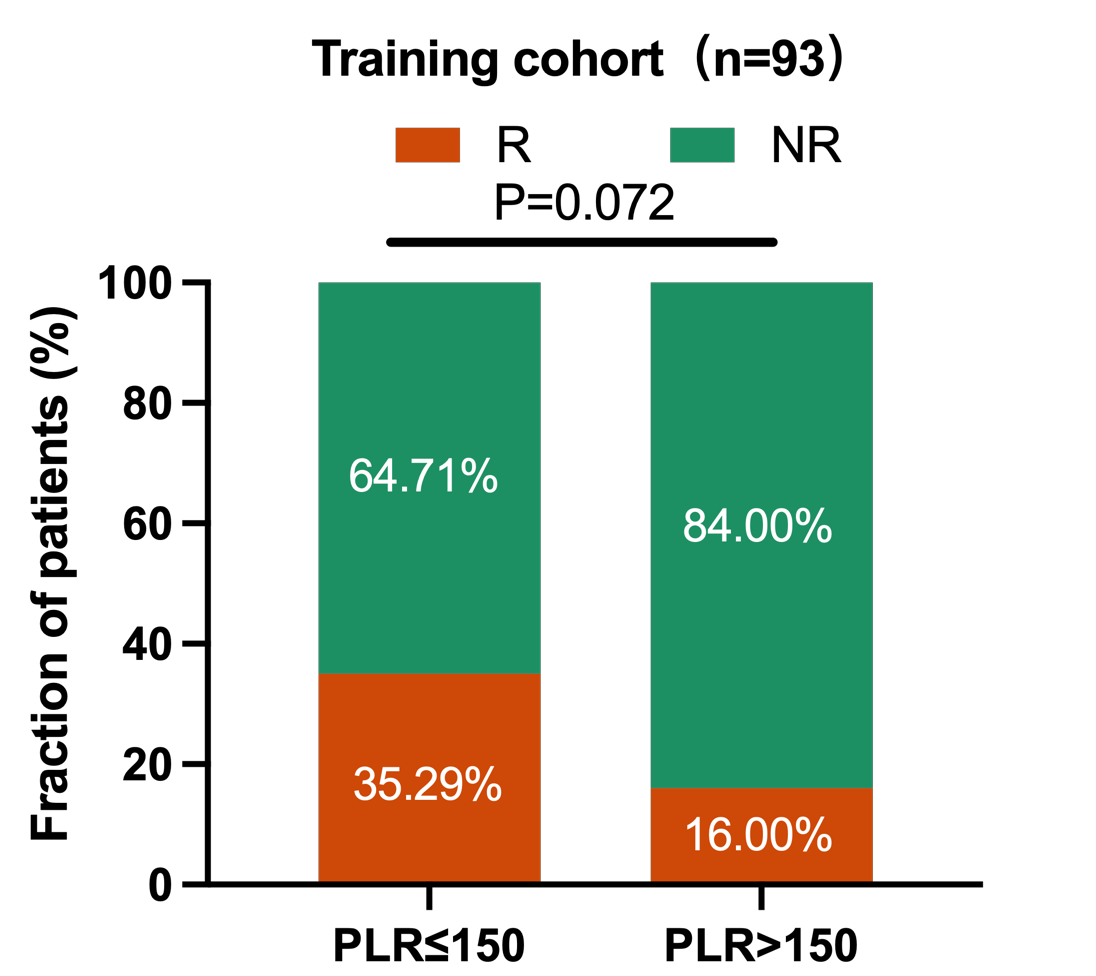


G H I J


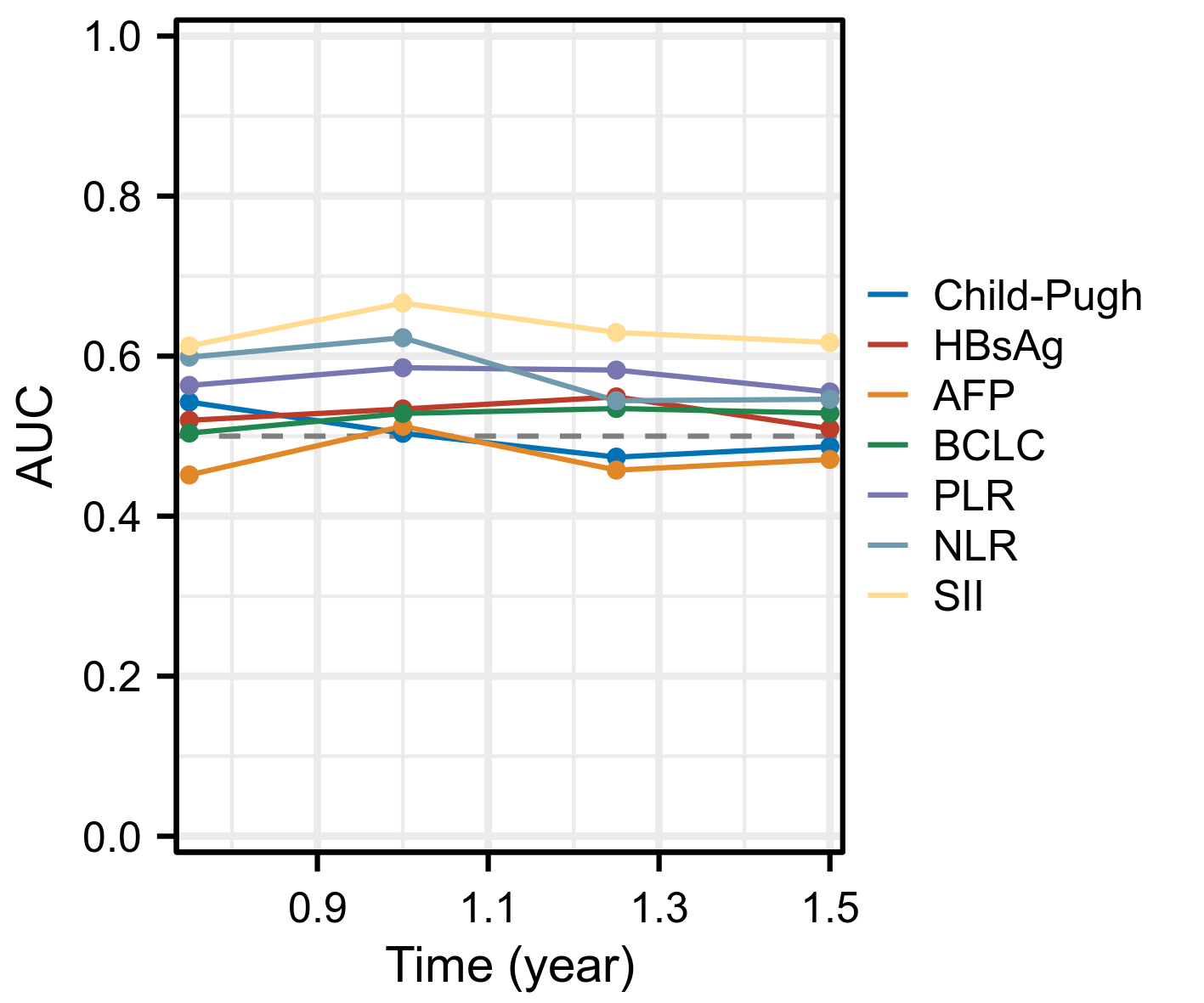

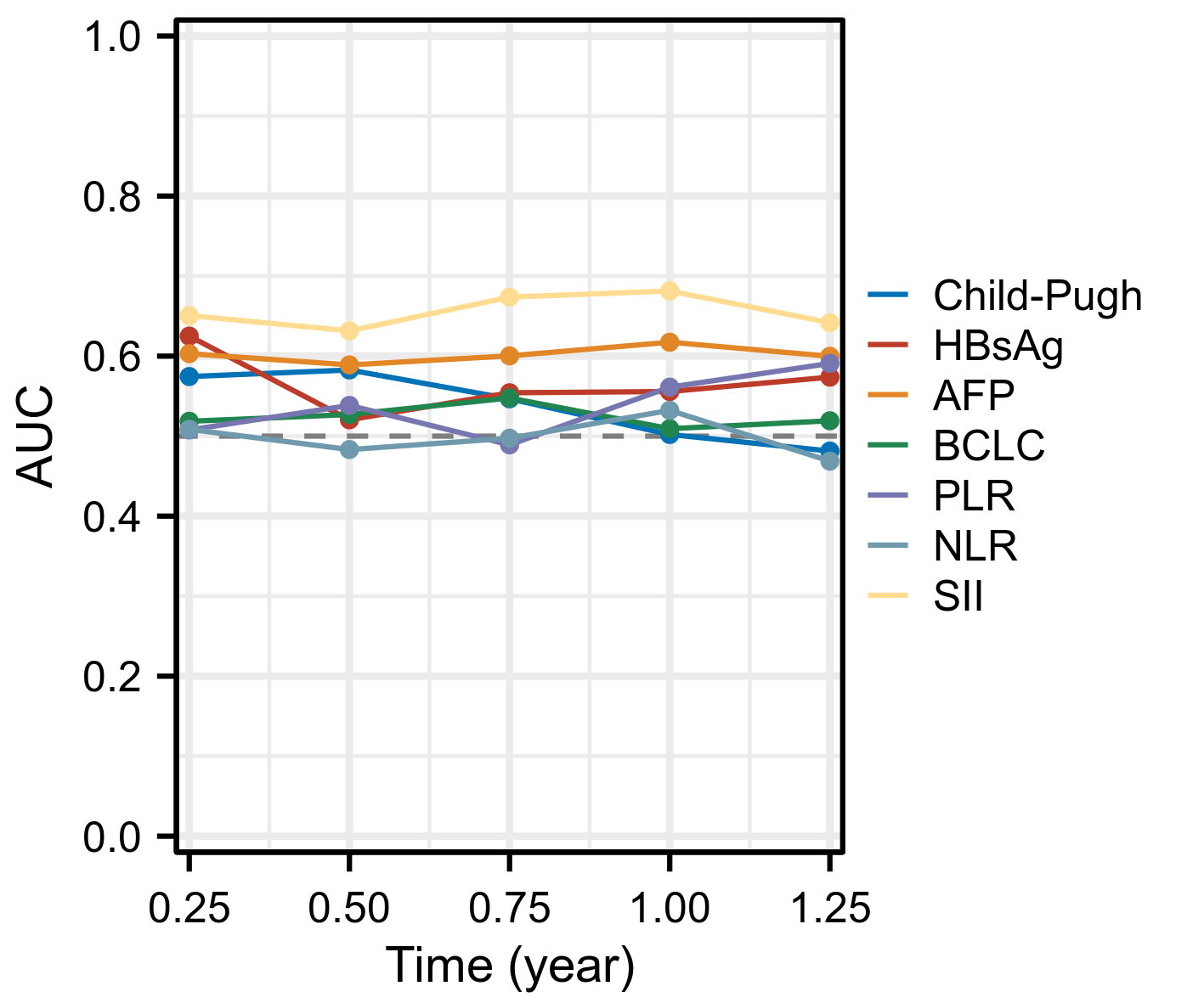

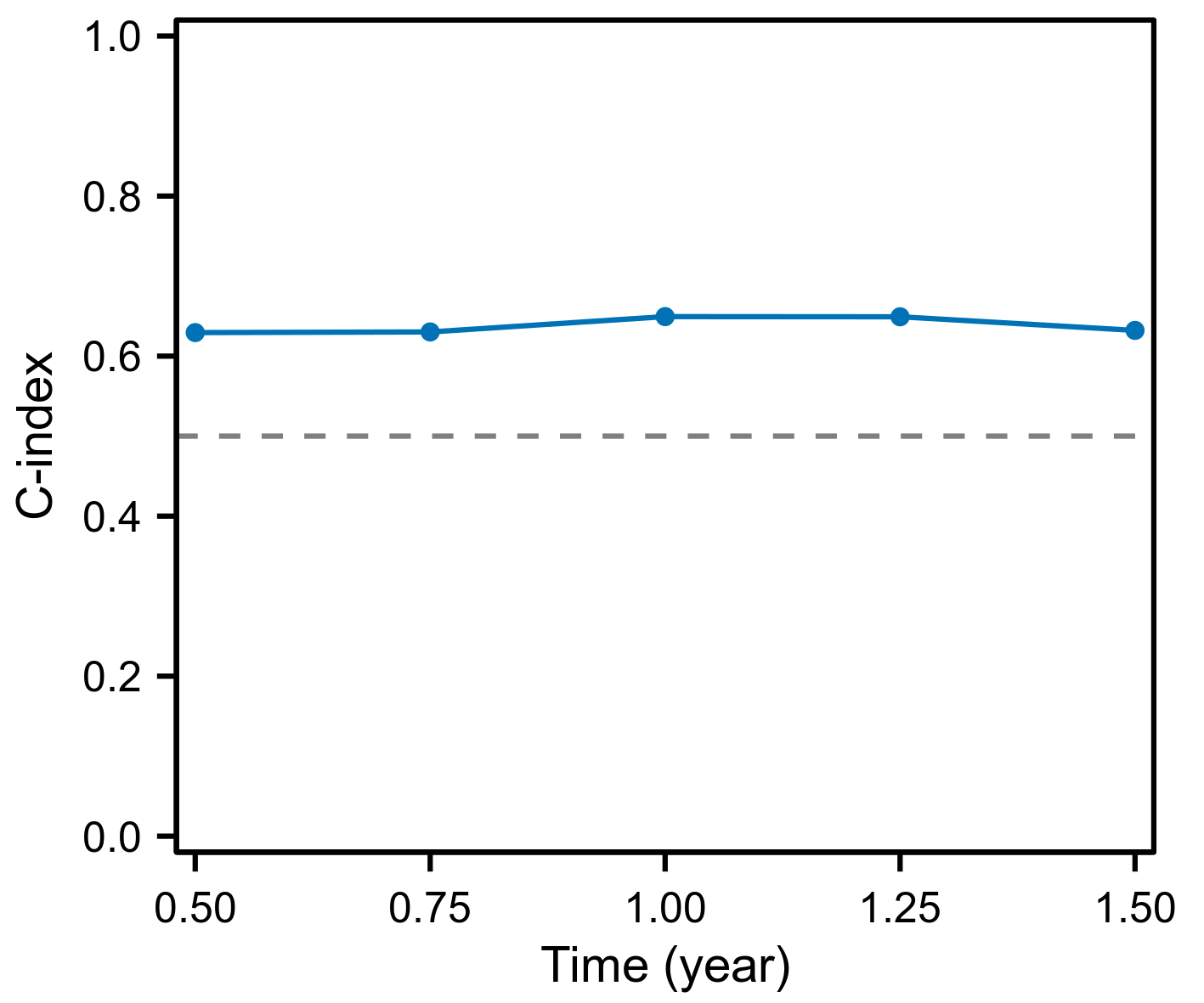

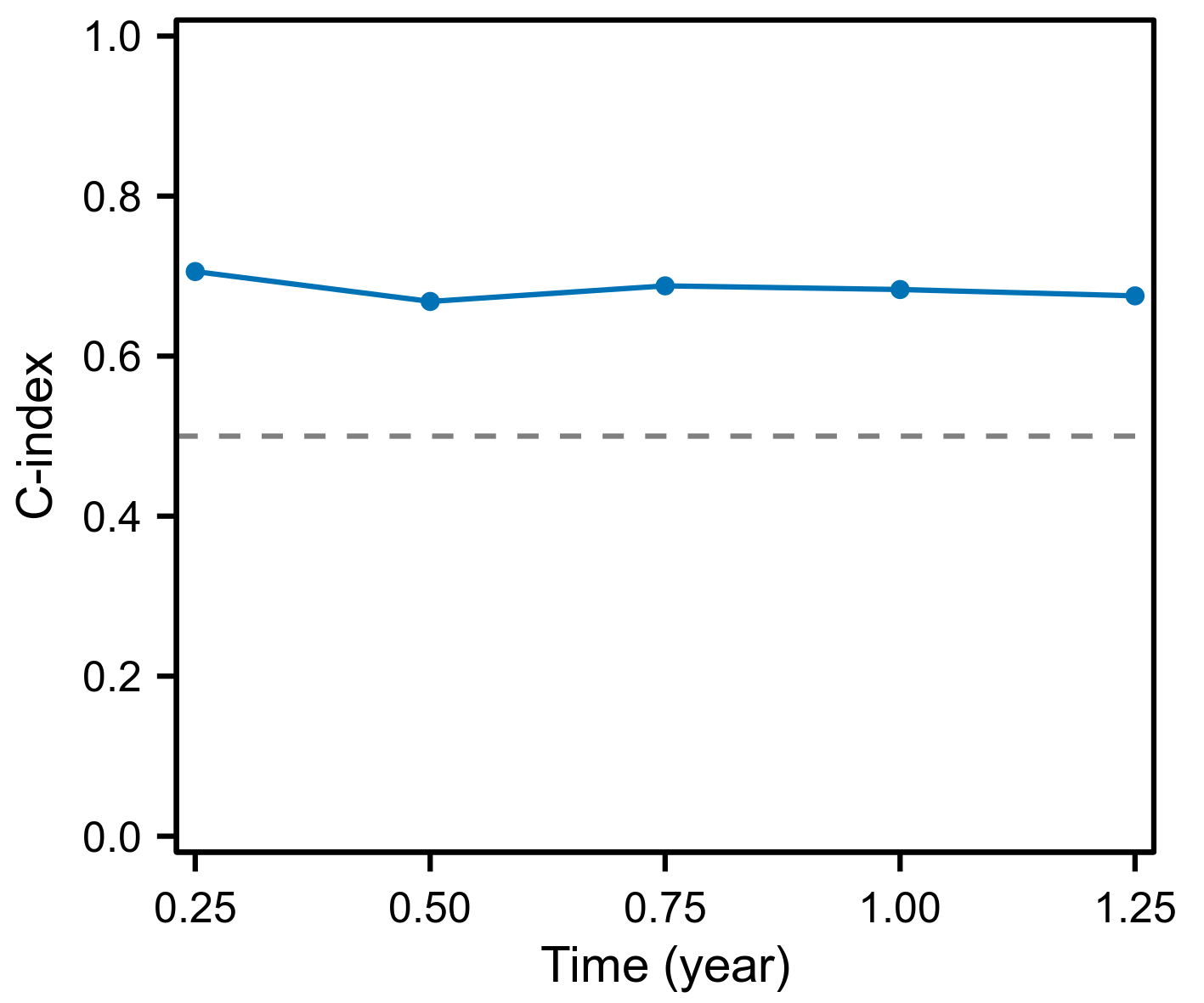


**Supplementary Figure S3.** The Kaplan–Meier analysis of PFS (A) and OS (B) for the PLR in the training cohort. The Kaplan–Meier analysis of PFS (C) and OS (D) for the NLR in the training cohort. Comparison of ORR for the NLR and PLR in the training cohort (E - F). Time-dependent AUC and the C-index of OS and PFS for SII in the training cohort(G - J).

**Supplementary Figure S4**

A B


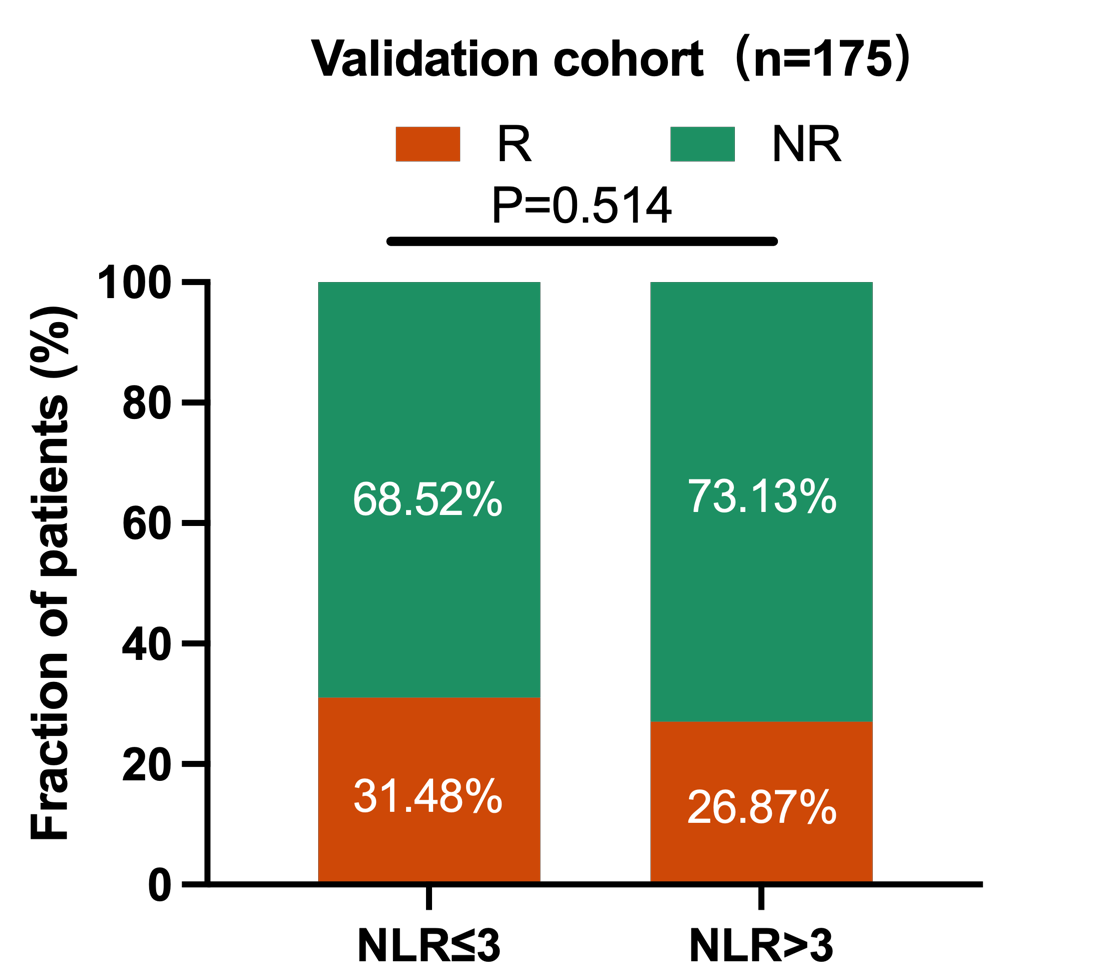

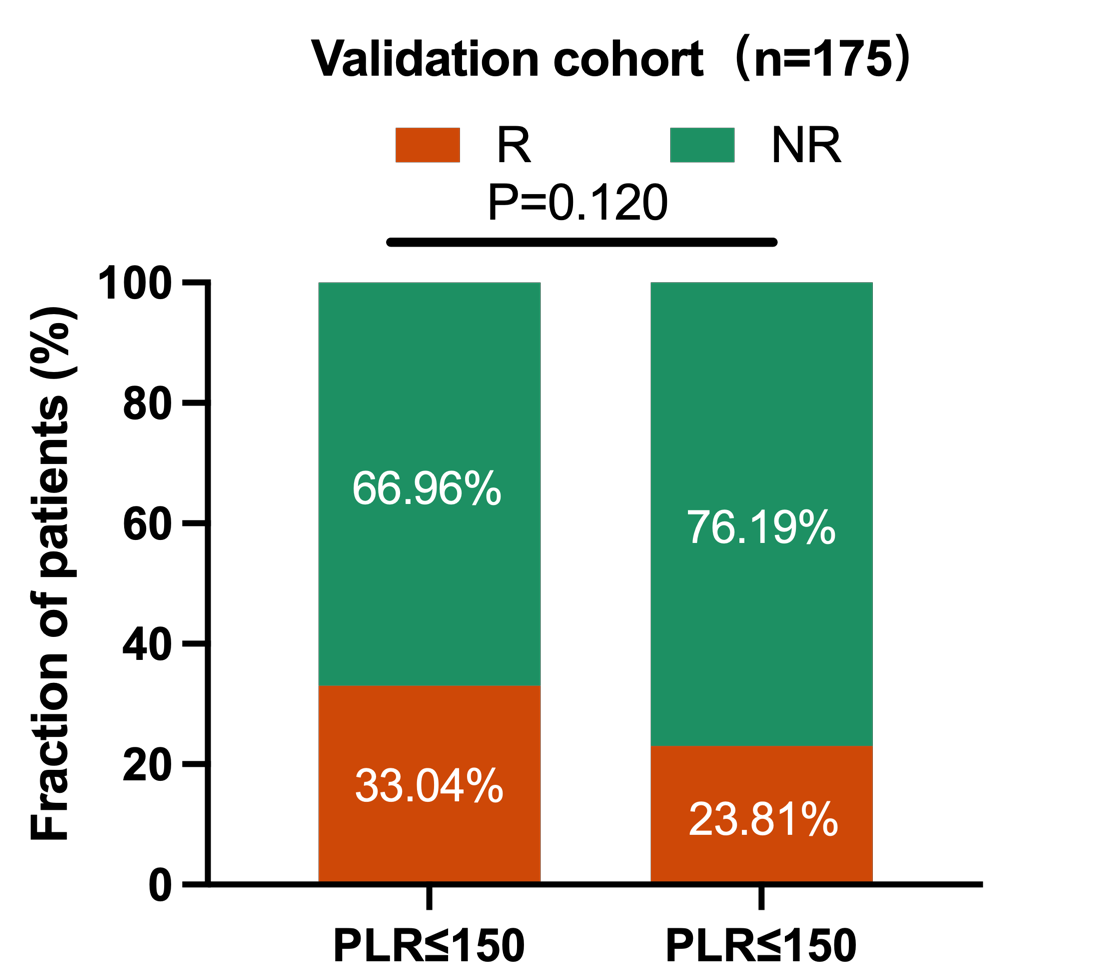


C D


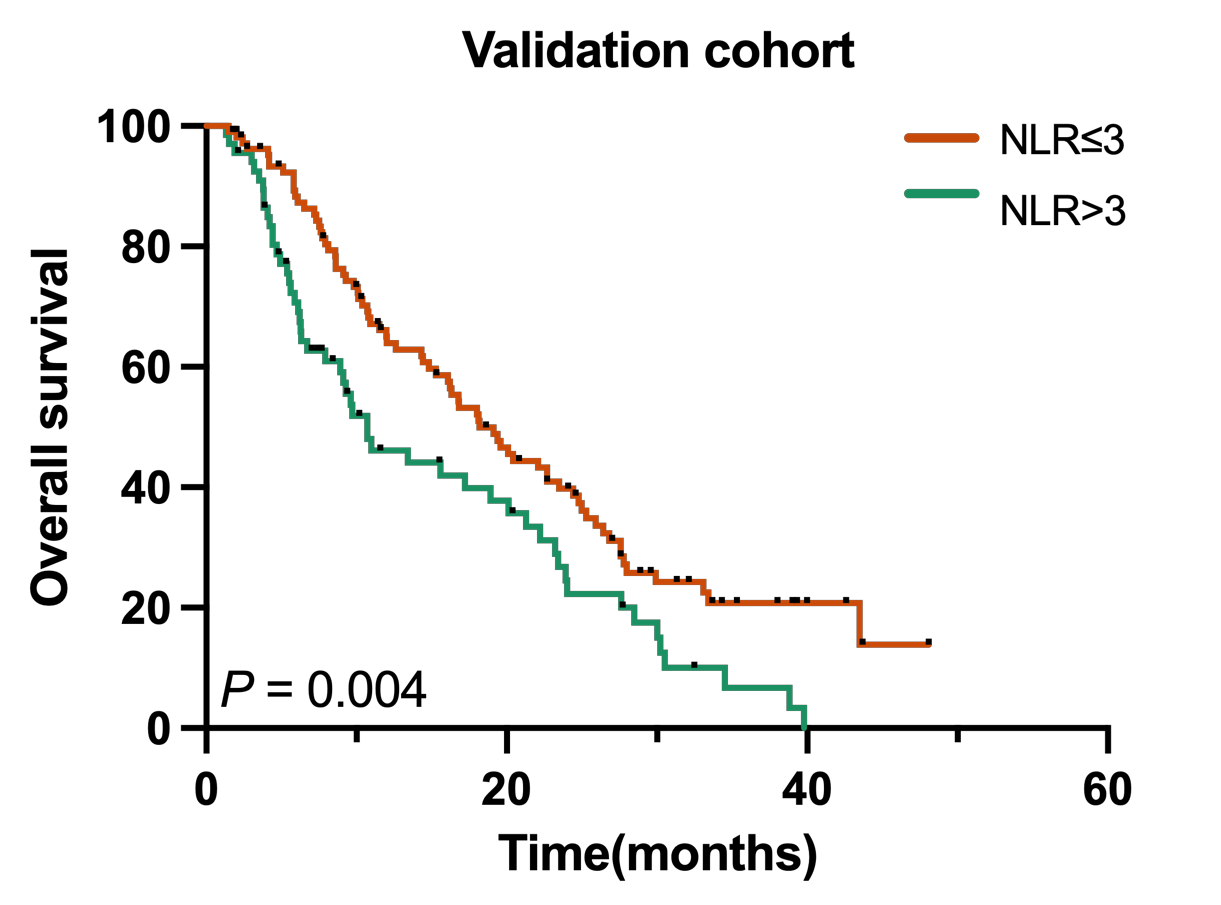

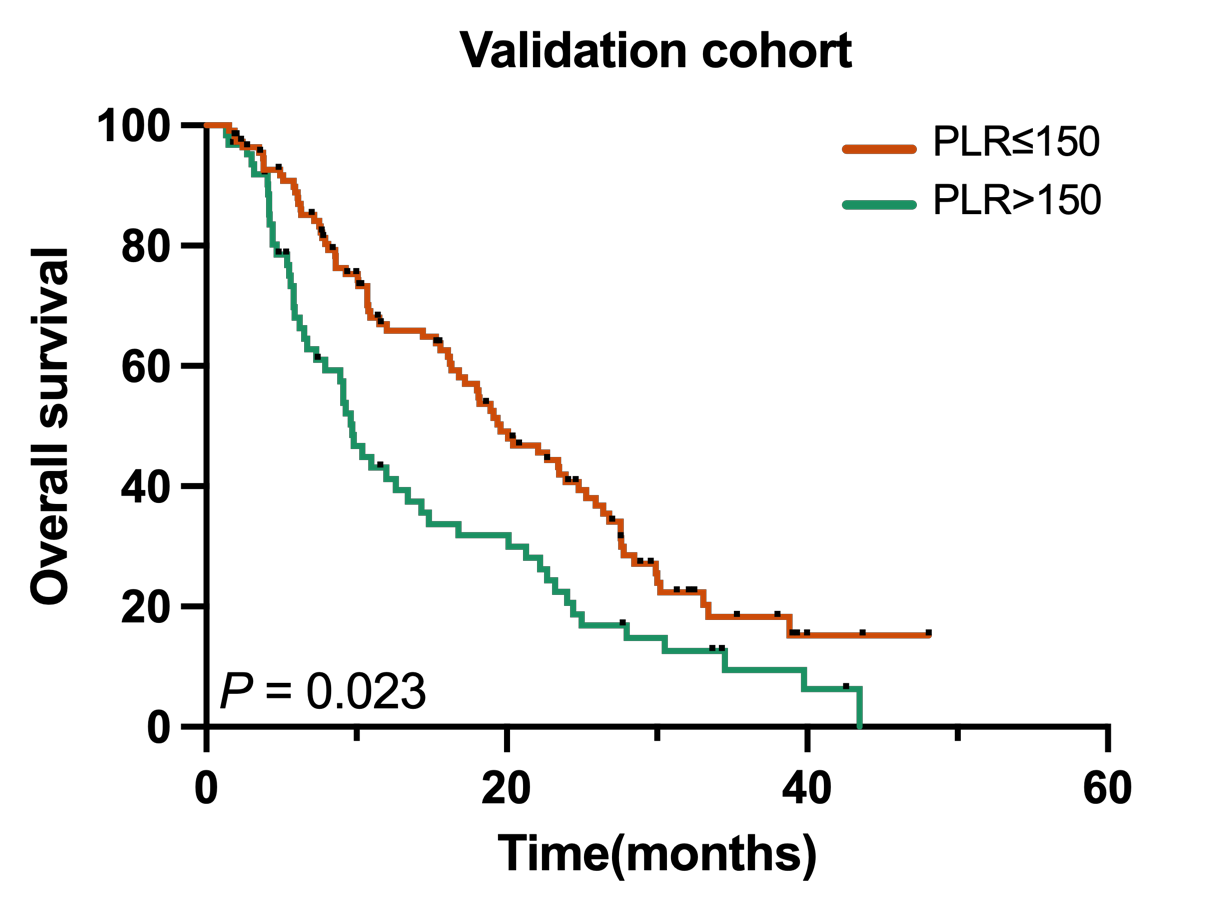


E F


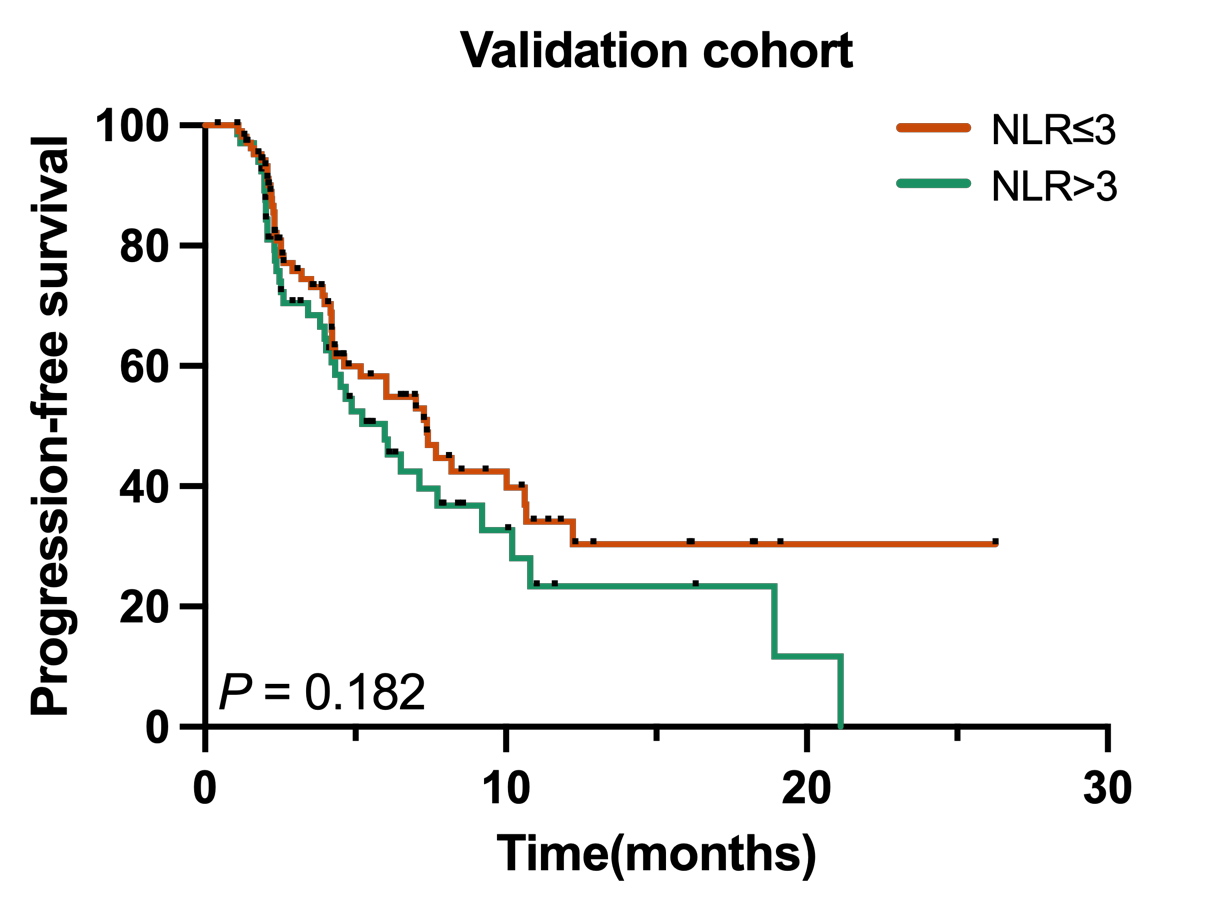

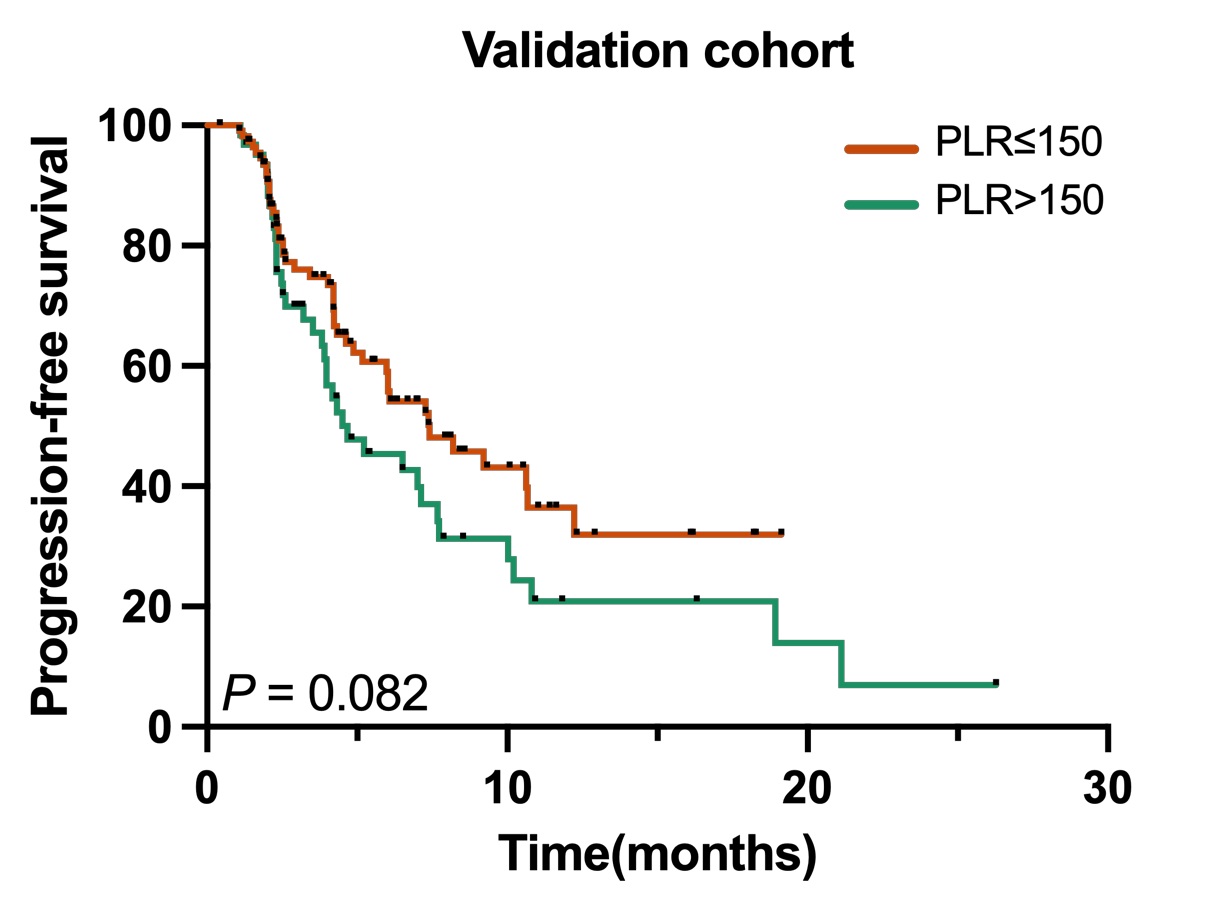


G H I J


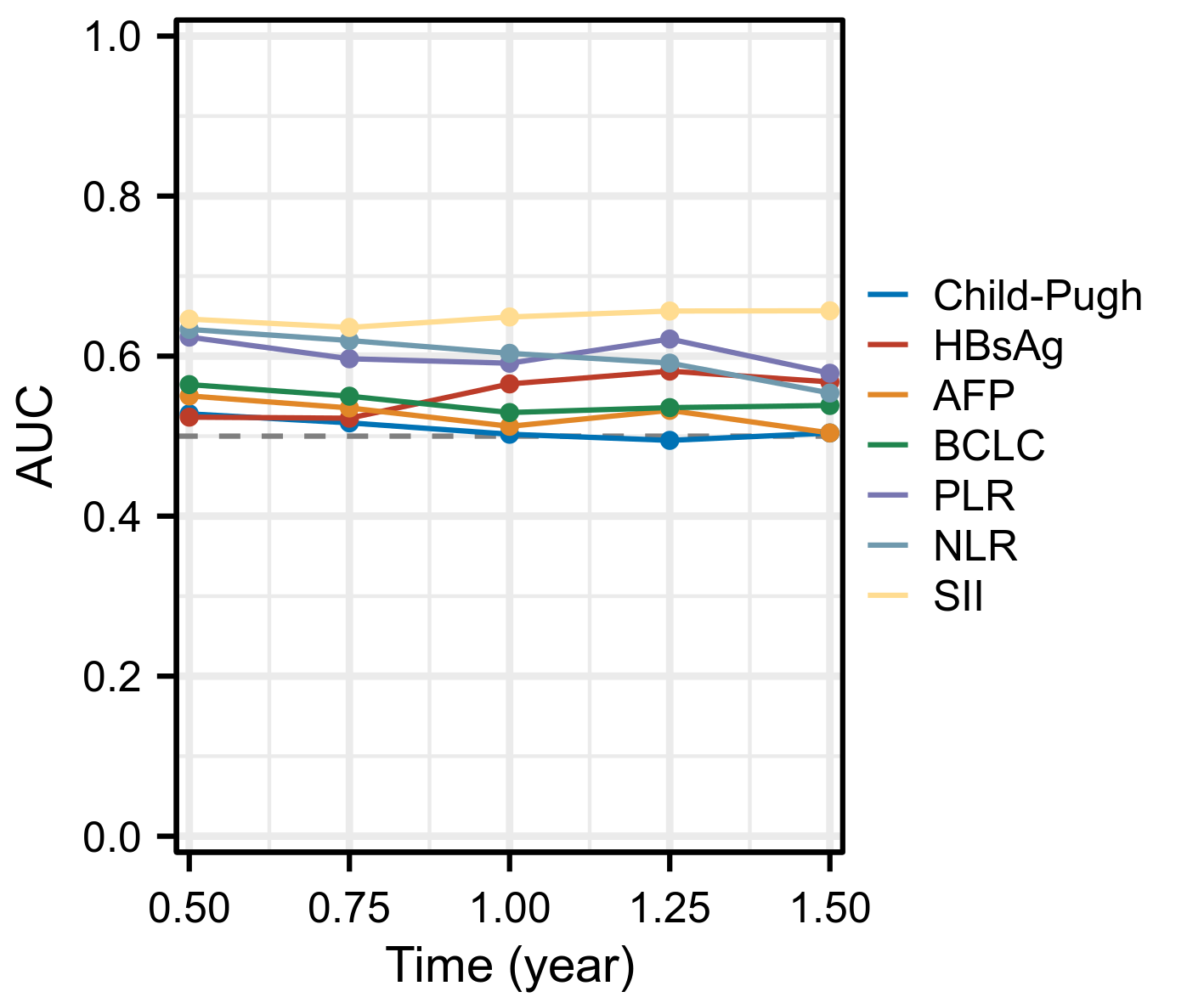

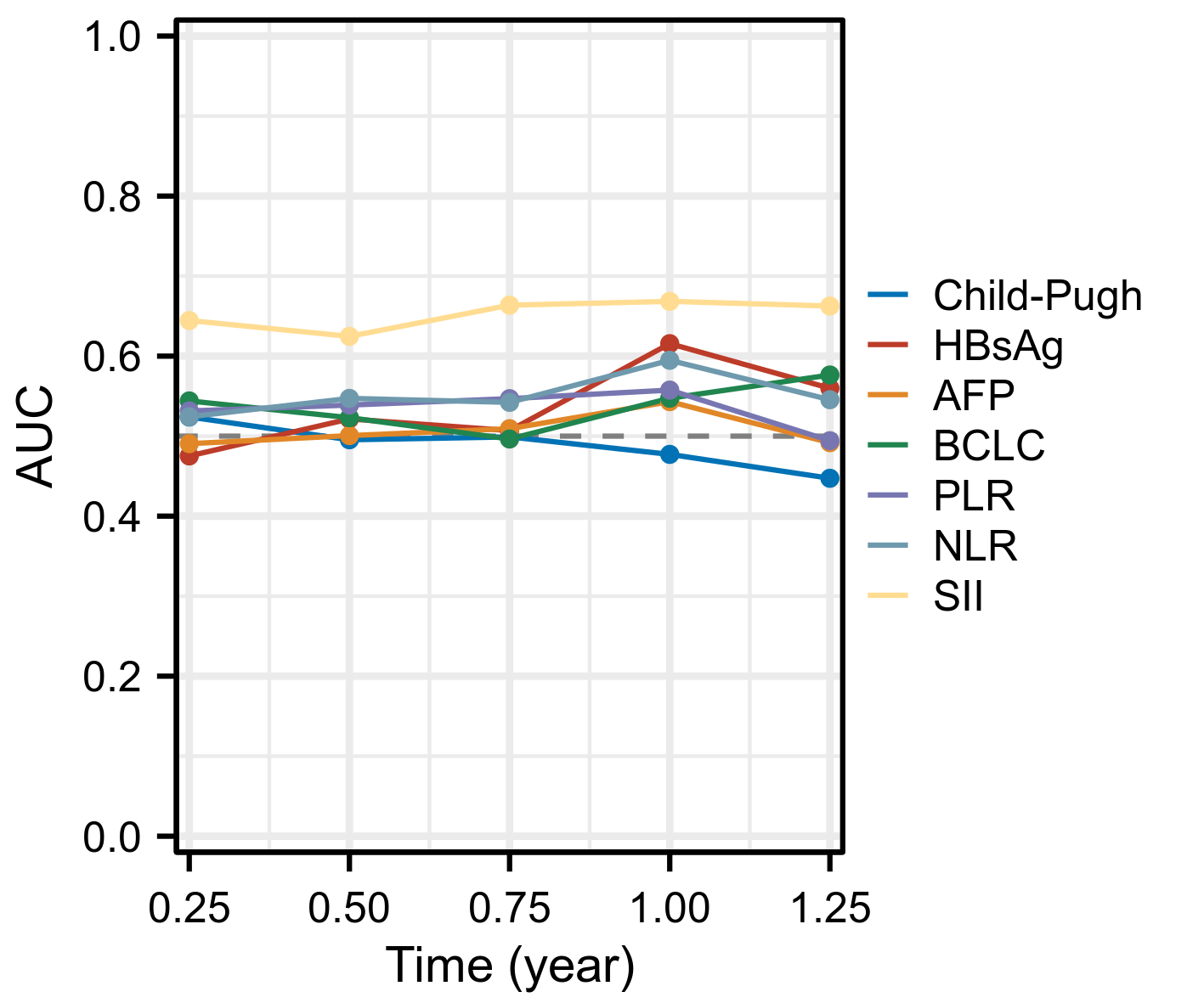

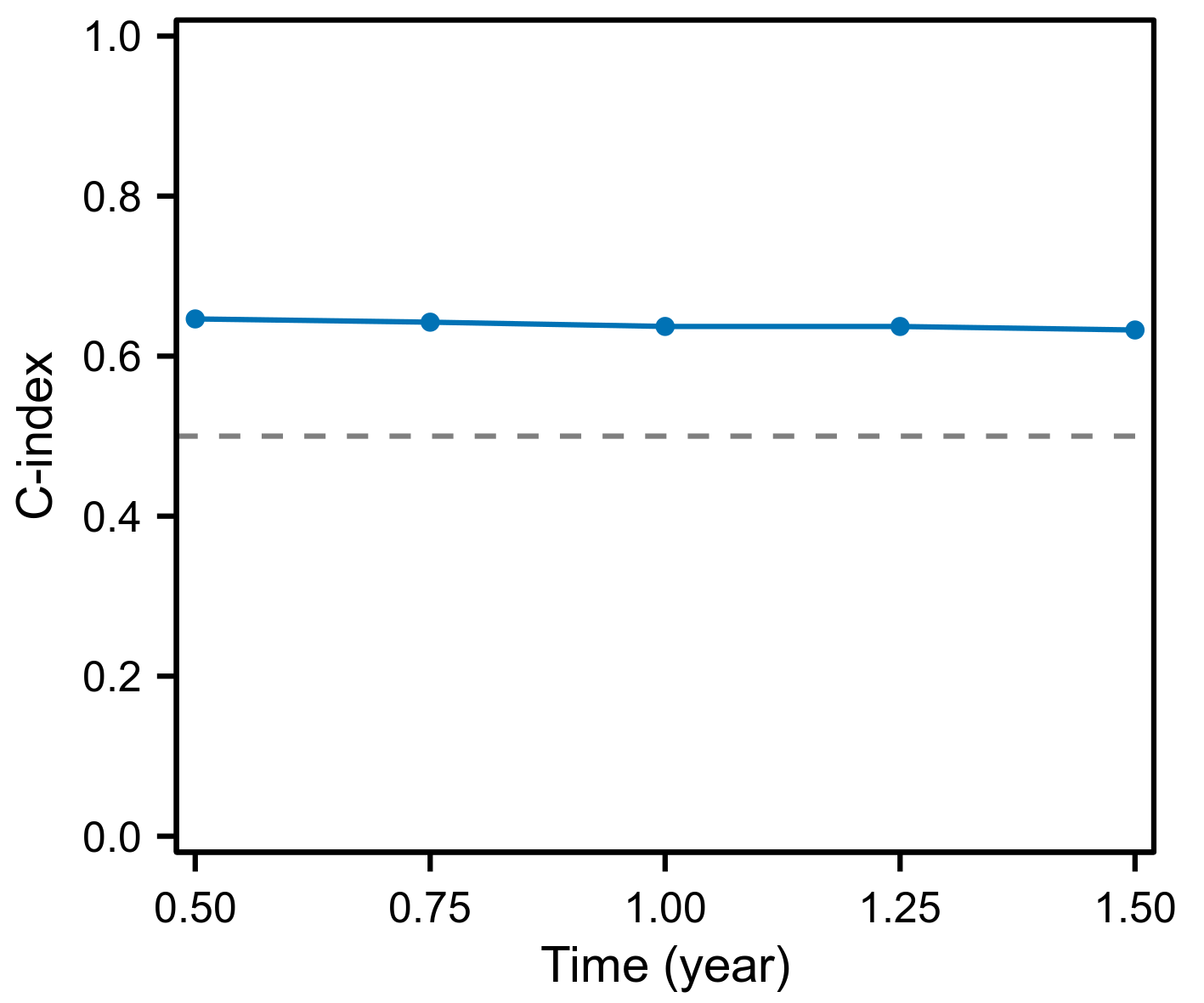

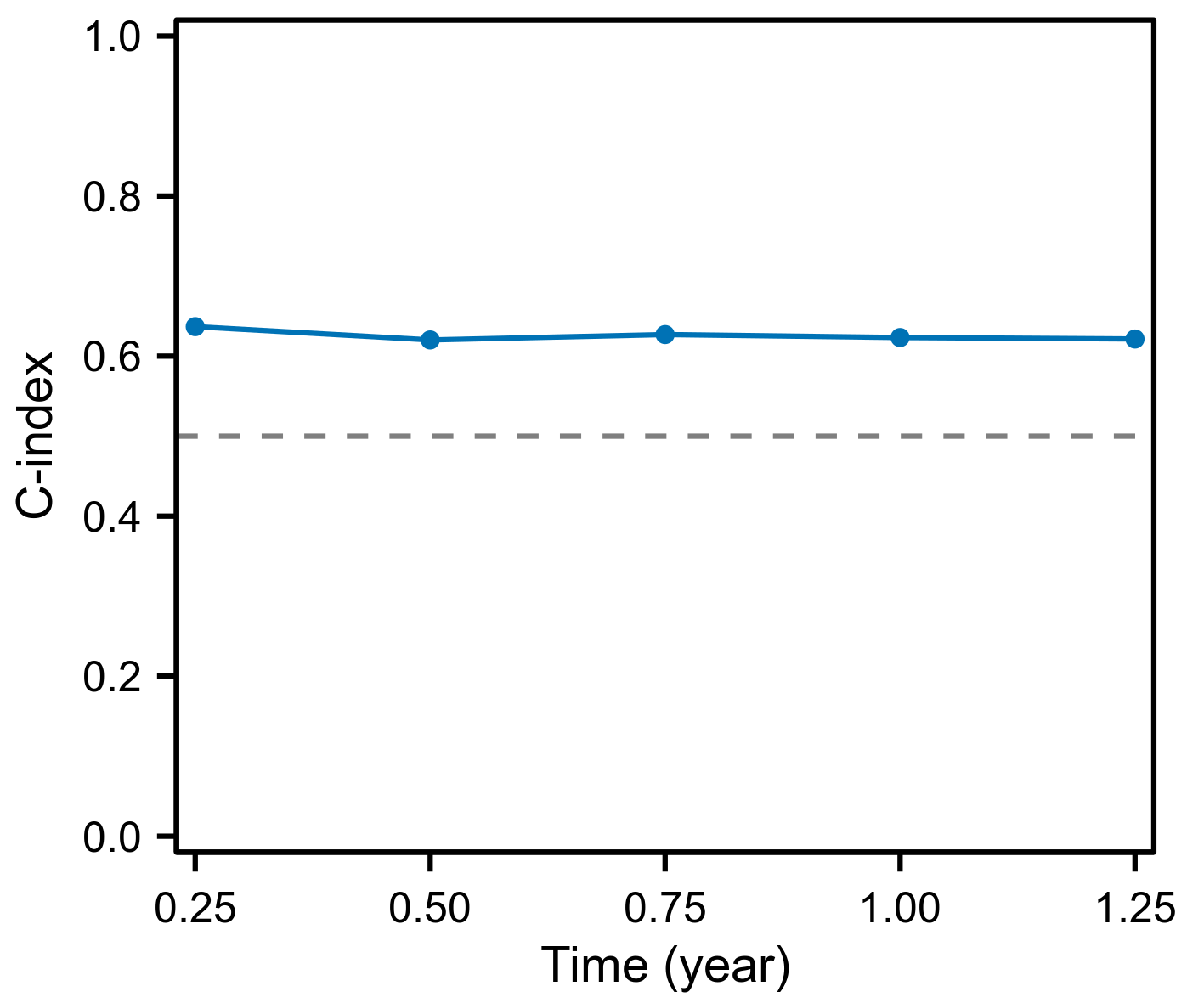


**Supplementary Figure S4.** Comparison of ORR for the NLR and PLR in the validation cohort (A-B). The Kaplan–Meier analysis of PFS and OS for the PLR and NLR in the validation cohort (C - F). Time-dependent AUC and the C-index of OS and PFS for SII in the validation cohort (G - J).

**Supplementary table S1.** Univariate and multivariate logistic regression analyses of prognostic factors for response probability in the training and validation cohort

| Variable | Category | Univariate analysis | | |  | Multivariate analysis |  |
| --- | --- | --- | --- | --- | --- | --- | --- |
|  |  | OR (95% CI) | P-value | |  | OR (95% CI) | P-value |
| Training cohort | | | | | | | |
| Age(y) | >50 vs. ≤50 | 1.156 (0.451 - 2.966) | | 0.763 |  |  |  |
| Sex | Male vs. Female | 2.000 (0.494 - 8.089) | | 0.331 |  |  |  |
| Child-Pugh | B vs. A | 1.322 (0.250 - 6.991) | | 0.743 |  |  |  |
| HBsAg | Positive vs. Negative | 1.639 (0.424 - 6.330) | | 0.474 |  |  |  |
| ALT(U/L) | >50 vs. ≤50 | 1.380 (0.448 - 4.256) | | 0.575 |  |  |  |
| AFP (ng/mL) | >20 vs. ≤20 | 1.182 (0.474 - 2.944) | | 0.720 |  |  |  |
| Macrovascular tumor thrombus | Yes vs. No | 1.193 (0.452 - 3.150) | | 0.721 |  |  |  |
| Extrahepatic metastasis | Yes vs. No | 1.432 (0.526 - 3.896) | | 0.482 |  |  |  |
| BCLC | B+C vs. A | 1.590 (0.251 - 10.079) | | 0.623 |  |  |  |
| NLR | >3 vs. ≤3 | 2.353 (0.787 - 7.036) | | 0.126 |  |  |  |
| PLR | >150 vs. ≤150 | 2.864 (0.881 - 9.312) | | 0.080 |  | 1.580 (0.418 - 5.973) | 0.500 |
| SII | >752 vs. ≤752 | 5.370 (1.158 - 24.901) | | 0.032 |  | 4.185 (0.778 - 22.515) | 0.095 |
| Validation cohort | | | | | | | |
| Age(y) | >50 vs. ≤50 | 1.667 (0.790 - 3.515) | | 0.180 |  |  |  |
| Sex | Male vs. Female | 1.174 (0.461 - 2.990) | | 0.736 |  |  |  |
| Child-Pugh | B vs. A | 1.439 (0.532 - 3.890) | | 0.473 |  |  |  |
| HBsAg | Positive vs. Negative | 1.508 (0.731 - 3.111) | | 0.266 |  |  |  |
| ALT(U/L) | >50 vs. ≤50 | 0.806 (0.385 - 1.685) | | 0.566 |  |  |  |
| AFP (ng/mL) | >20 vs. ≤20 | 1.705 (0.858 - 3.386) | | 0.128 |  |  |  |
| Macrovascular tumor thrombus | Yes vs. No | 1.089 (0.567 - 2.090) | | 0.798 |  |  |  |
| Extrahepatic metastasis | Yes vs. No | 1.292 (0.675 - 2.474) | | 0.439 |  |  |  |
| BCLC | B+C vs. A | 1.418 (0.440 - 4.573) | | 0.559 |  |  |  |
| NLR | >3 vs. ≤3 | 1.251 (0.636 - 2.458) | | 0.516 |  |  |  |
| PLR | >150 vs. ≤150 | 1.859 (0.913 - 3.784) | | 0.087 |  | 1.336 (0.599 - 2.978) | 0.479 |
| SII | >752 vs. ≤752 | 2.660 (1.097 - 6.452) | | 0.030 |  | 2.265 (0.842 - 6.094) | 0.106 |

Abbreviation: ALT, albumin; AFP, alpha‐fetoprotein; BCLC, Barcelona Clinic Liver Cancer; NLR, neutrophil-to-lymphocyte ratio; PLR, platelet-to-lymphocyte ratio; SII, systemic immune-inflammation index
